# The outer mucus layer hosts a distinct intestinal microbial niche

**DOI:** 10.1038/ncomms9292

**Published:** 2015-09-22

**Authors:** Hai Li, Julien P. Limenitakis, Tobias Fuhrer, Markus B. Geuking, Melissa A. Lawson, Madeleine Wyss, Sandrine Brugiroux, Irene Keller, Jamie A. Macpherson, Sandra Rupp, Bettina Stolp, Jens V. Stein, Bärbel Stecher, Uwe Sauer, Kathy D. McCoy, Andrew J. Macpherson

**Affiliations:** 1 Maurice Müller Laboratories (DKF), Universitätsklinik für Viszerale Chirurgie und Medizin Inselspital, University of Bern, Murtenstrasse 35, 3010 Bern, Switzerland; 2 Institute of Molecular Systems Biology, Swiss Federal Institute of Technology (ETH) Zürich, Auguste-Piccard-Hof 1, 8093 Zürich, Switzerland; 3 Max-von-Pettenkofer Institute, German Center for Infection Research (DZIF), Pettenkoferstrasse 9a, Partner site LMU Munich, D-80336 Munich, Germany; 4 Theodor Kocher Institute, Freiestrasse 1, University of Bern, 3012 Bern, Switzerland

## Abstract

The overall composition of the mammalian intestinal microbiota varies between individuals: within each individual there are differences along the length of the intestinal tract related to host nutrition, intestinal motility and secretions. Mucus is a highly regenerative protective lubricant glycoprotein sheet secreted by host intestinal goblet cells; the inner mucus layer is nearly sterile. Here we show that the outer mucus of the large intestine forms a unique microbial niche with distinct communities, including bacteria without specialized mucolytic capability. Bacterial species present in the mucus show differential proliferation and resource utilization compared with the same species in the intestinal lumen, with high recovery of bioavailable iron and consumption of epithelial-derived carbon sources according to their genome-encoded metabolic repertoire. Functional competition for existence in this intimate layer is likely to be a major determinant of microbiota composition and microbial molecular exchange with the host.

## Abstract (web summary)

The inner layer of the mucus that covers our intestine is nearly sterile. Here, the authors show in mice that the outer mucus layer constitutes a unique microbial niche hosting bacterial communities with distinct proliferation rates and resource utilization activities.

## Graphical abstract

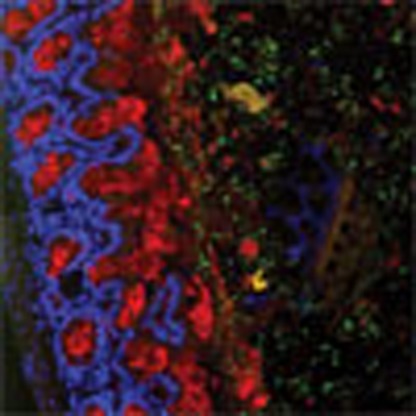

Mucus is known to be a highly dynamic matrix, largely consisting of mucin glycoproteins sheets, secreted by intestinal goblet cells, which lubricates the transit of intestinal contents. In the small intestine the mucus is discontinuous, but in the stomach and large intestine (colon) there are two layers^1^. Tight stacking of polymeric glycoproteins adjacent to the epithelium forms a compact inner layer that is largely sterile^2^. Following proteolytic dispersion of mucin polymers, the outer layer is looser and contains intestinal bacteria^1^. Goblet cell secretion of mucin is orchestrated by inflammasome activity^3^, and mucus thickens as the microbiota becomes more diverse^4^.

Mucus therefore forms an important intestinal compartment, and the glycoprotein is itself a microbial carbon source. Only some bacterial species have a sufficient repertoire of genome-encoded catabolic glycosidic enzymes to disassemble complex mucus glycans as a sufficient carbon source, so relatively few microbes can be considered as mucolytic specialists. For these mucolytic bacteria, mucus is a potentially distinct microbiological niche on the basis of the available carbon source. Mucus oxygen levels at atmospheric pressures are also low enough to allow the presence of anaerobes^5^. For example, *Bacteroides thetaiotaomicron* has been shown to forage on mucus glycans in the caecum when plant polysaccharides are absent from the diet^6^,^7^. The ability of *B. thetaiotaomicron* in the caecum to use mucus as an alternative carbon source is consistent with the concept that the outer layer of mucus is a separate microbiological niche for such mucolytic bacteria. However, this interpretation is indirect, because mucus is being constantly shed into the intestinal lumen^2^, so overall metabolic alterations under conditions of nutrient restriction do not necessarily show the extent of differences in resource utilization between the outer mucus layer and the intestinal contents under normal physiological and dietary conditions.

Aside from the utilization of mucus glycans as a niche carbon source, mucus is also known to retain non-mucolytic bacterial species^8^,^9^. For example, commensal *Escherichia coli* was found to replicate preferentially in the mucus during regrowth after antibiotic treatment on the basis of ribosomal content assessments^10^.

If the outer mucus layer is a generally separate microbiological niche for mucolytic non-specialists and specialists alike, three outstanding questions need to be addressed^11^,^12^,^13^. (i) Is the outer mucus layer a physical habitat sufficiently separate for microbes to assemble into distinct communities? (ii) Is there differential resource utilization of constituents other than mucin glycoprotein or oxygen? (iii) Do microbes in the mucus adopt special strategies for population persistence, such as general transcriptional and metabolic differences, compared with the same species in an adjacent compartment (in this case, the intestinal lumen)?

There is certainly evidence of minor differences between the composition of microbial communities in the outer layer of mucus of the large intestine and the luminal intestinal contents. This is on a background of considerable variability between the microbiota of mouse colonies, even between different rooms of the same vivarium, that changes the structure and functional properties of the mucus layer^4^. The dynamics of rapid mucus shedding into the lumen^14^ also mean that luminal bacteria will inevitably be contaminated with any bacterial species that have replicated in the mucus layer, so composition assessments *per se* do not reveal whether there are metabolic patterns that potentially define the general differences within individual species in resource utilization or strategies for population persistence within the mucus compared with the intestinal contents.

The very intimacy between microbial populations in the outer mucus layer and host intestinal tissues makes the existence of an outer mucus niche of intestinal microbes extremely important, as microbial metabolites are known to determine the differentiation and function of epithelial and immune cells in the intestinal mucosa^15^,^16^. Put the other way around, the host intestinal epithelial layer is being constantly shed^14^, giving scope for the host to succor different microbial species in their race to replicate and avoid extinction from the microbiota.

In this paper, we analyse the spatial distribution, functional metabolic and proliferative adaptation and fitness of different intestinal bacteria in the outer layer of colonic mucus in simple defined microbiotas. To understand the scope of niche differences in these parameters, we analyse the contrasting situations of monocolonization with two genetically well-defined intestinal microbes: *B. thetaiotaomicron* (a mucolytic symbiont, which is an abundant member of the healthy intestinal microbiota^6^,^7^) and *E. coli* (a minor constituent that blooms under conditions of infectious and non-infectious inflammation with limited glycoside hydrolase activity for mucus metabolism^17^,^18^,^19^,^20^,^21^,^22^). In these defined model gnotobiotic mice, we show that the outer mucus niche concept applies to resource utilization ranging beyond mucus metabolism, to mineral harvesting and utilization of shed host phospholipids. These differences in resource utilization reflect the specific bacterial genomic repertoires and establish distinct strategies for population persistence in the mucus compared with the luminal intestinal contents under normal fed physiological conditions.

## Results

### Microbial consortia in the outer mucus layer

**Figure 1:**
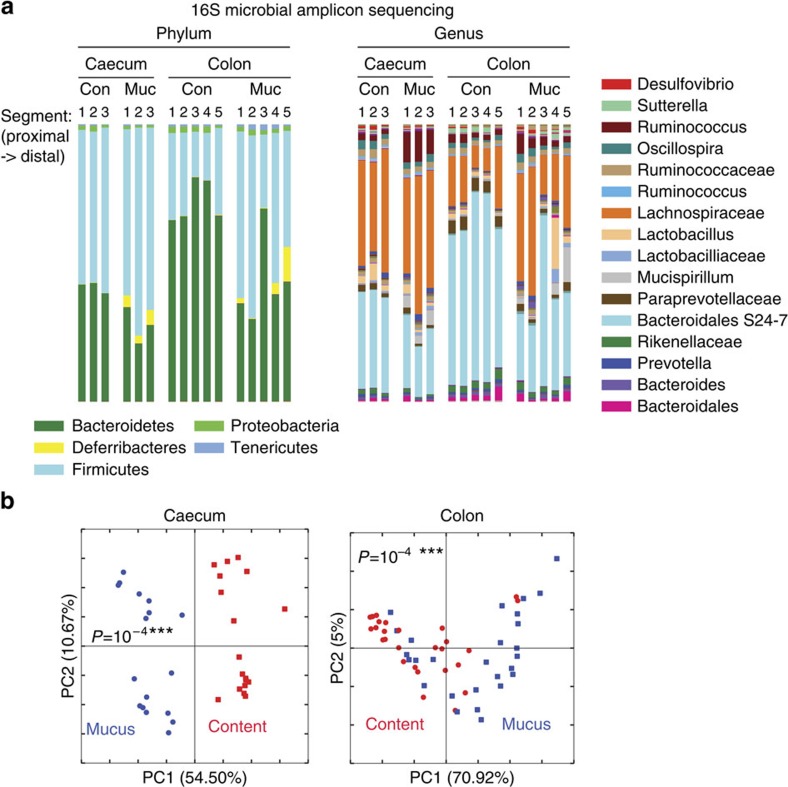
Microbial communities in the mucus layer and content vary in the intestine. (**a**) The microbial composition within the mucus layer (Muc) and luminal content (Con) of different segments along the caecum and colon of SPF mice was determined by 16S amplicon analysis. Representative bar graphs from one mouse out of four to six mice per group are shown. (**b**) Principal coordinates analysis on weighted UniFrac distances was performed on all operational taxonomic units. *P*-values to determine the statistical significance of clustering were calculated using the Adonis method. Analysis was performed using QIIME 1.8.0.

To determine how far the compositions of microbial populations differ according to transverse compartmentalization (within the mucus or the luminal contents) in the intestine, we started by comparing the compositions of the microbiota in aspirates of the outer mucus layer with those in the intestinal lumen of C57BL/6 wild-type mice colonized with a diverse specific pathogen-free (SPF) microbiota consisting of operational taxonomic units that cover more than 100 different genera (colonic luminal Shannon alpha diversity 8.22±0.88, 
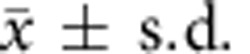
, *n*=28, Supplementary Fig. 1) We concentrated the analysis on the large intestine, because this is where the mucus layer is thickest and the microbial concentration is highest (refs 1, 23 and Supplementary Fig. 2). We also confirmed by immunofluorescent staining and by using green fluorescent protein (GFP)-expressing bacteria with two-photon microscopy at different *Z*-planes in these simplified animal models that bacteria were resident in the outer mucus layer (Supplementary Fig. 3). In the caecum and colon of SPF animals, high-throughput 16S amplicon sequencing (Fig. 1a) and principal coordinate analysis (PCoA) on weighted Unifrac distances (Fig. 1b) showed that the outer mucus layer had different representations of microbes compared with the intestinal lumen, and no significant differences were seen longitudinally along the gut (Supplementary Fig. 4). Previous studies have also found differences in the composition of mucus and luminal intestinal microbial compositions of diverse microbiotas, for example, with *Ruminococcus* species, although considerable variability has been observed between colonies, even when housed in different rooms of the same vivarium^4^.

To confirm these data in a system where we have information at the species level, we generated a gnotobiotic mouse model with a stable defined moderately diverse mouse microbiota (sDMDMm_2_) that consists of 12 bacterial species (colonic luminal Shannon diversity 1.98±0.38, 
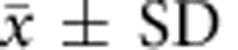
, *n*=11, Supplementary Fig. 1). Using these stable isobiotic mice, 16S amplicon and PCoA also showed that the compositions in the large intestinal outer mucus and the intestinal lumen were significantly different, although all constituents of the isobiotic microbiota were consistently present at some level in both compartments (Supplementary Fig. 5). A limitation in applying microbiota composition analysis alone to substantiate the recess concept of a separate microbiological niche in the outer mucus layer compared with the luminal contents is that functional differences may be underestimated. Rapid turnover of mucus (ref. 14 and Supplementary Fig. 6a) causes shedding of its microbial constituents, which are then detected in the intestinal lumen, even though they may not form a stable regenerative community in the luminal contents.

A generally distinct niche in the outer mucus would imply that bacterial mobility is restricted, even where the strain is motile. To assess this, we studied C57BL/6 mice that had been monocolonized with a motile strain of *E. coli* (Supplementary Fig. 6b–d). Two-photon imaging confirmed that the bacteria were immobile up to the 5 min recording time in the outer mucus, whereas tumbling was observed in PBS media and swimming in luminal intestinal fluid (Supplementary Movies 1–3). This does not exclude a slower exchange between luminal and mucus compartments over the period of hours required for mucus regeneration and we cannot exclude the possibility that lack of visualized microbial motility in the mucus compartment could be due to technical imaging factors such as coverslip compression or the preparation of the tissue for imaging. Nevertheless, the morphology of the bacteria argued for replication rather than replacement as the mechanism of sustaining the mucus *E. coli* population in this monocolonized system. The mucus contained elongated bacilli and some forms at the end stage of binary fission (Supplementary Fig. 6e and f). This provided the first of three lines of evidence that, at least for *E. coli*, regeneration of the population in the mucus layer during mucus turnover occurs selectively through mucus bacterial cell division rather than repopulation from the luminal contents.

### Distinct transcriptional profiles of mucus layer microbes

Distinct bacterial niches in the outer mucus layer and the intestinal luminal contents also would imply that these are separate metabolic compartments with differential resource utilization, including, but not limited to, host mucus glycan constituents or increased oxygen availability in the mucus as an electron acceptor^5^. To address potential niche compartmentalization of bacterial metabolism, we initially studied C57BL/6 wild-type mice monocolonized either with *B. thetaiotaomicron* or *E. coli*. Both monocolonizations are rapidly progressing, achieving a high steady-state biomass of the monocolonizing bacterium within 18 h following a calculated inoculation dose of as little as 1–10 bacteria (Supplementary Fig. 7 and Supplementary Table 1). *B. thetaiotaomicron* is also known to be capable of both mucus and dietary glycan breakdown^7^,^24^, whereas *E. coli* has a restricted glycoside hydrolase repertoire (Supplementary Fig. 8), making them suitable contrasting models to study differential resource utilization under conditions where the mucus architecture measurements were not significantly different from mice permanently colonized for over 30 generations with an altered Schaedler flora (ASF; Supplementary Fig. 2b). These are simplified models, and the mucus structure does thicken in mice with very diverse colonization as shown for SPF animals (Supplementary Figs 1 and 2b). These monocolonizations did allow us to compare bacterial densities, transcriptional activities and metabolism in separate compartments for each individual microbial species independently of cross-feeding events, competition or inter-microbial exchange of vitamins, minerals or electron donors/acceptors, which can occur in mice that are colonized by multiple organisms.

**Figure 2:**
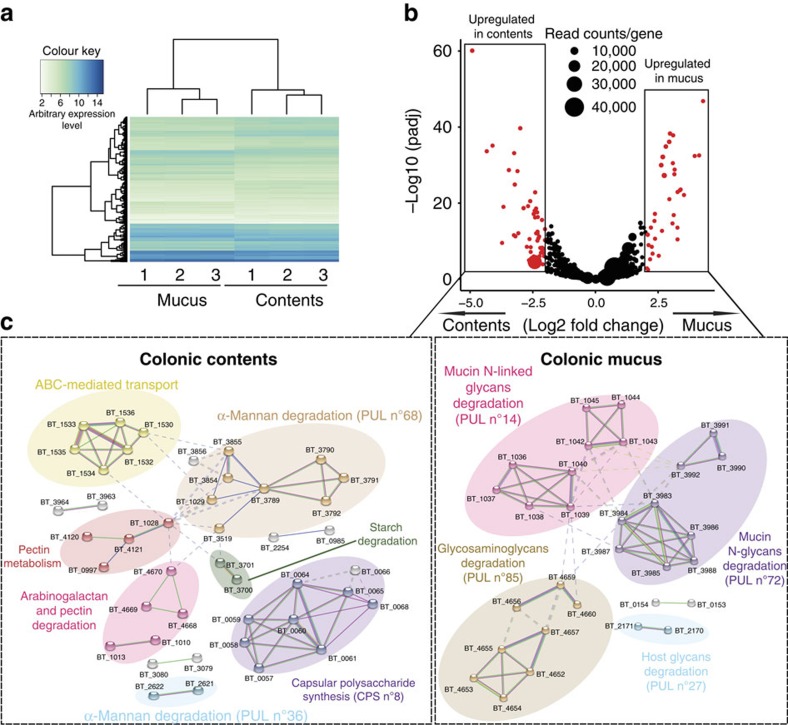
*B. thetaiotaomicron* in colonic mucus and contents has different transcriptional patterns. (**a**) A heat map of gene expression values shows differentially expressed genes identified by RNA-seq in bacteria isolated from colonic mucus and contents of *B. thetaiotaomicron* monocolonized C57BL/6 mice. Data shown are three experimental repeats from the two compartments. (**b**) The gene expression levels of *B. thetaiotaomicron* in colonic mucus were compared with colonic contents and the log2 transformed fold change was plotted against log10 transformed *P*-value adjusted (padj). Red-coloured dots indicate genes with log2 FoldChange ≥2 and padj ≤0.05. (**c**) A network analysis was performed on genes highlighted in red in **b** according to translated protein–protein interactions using STRING database. The formed gene clusters were differentially coloured and functional definitions were provided to according clusters. Original transcriptomic data with annotations are given in Supplementary Data 1.

**Figure 3:**
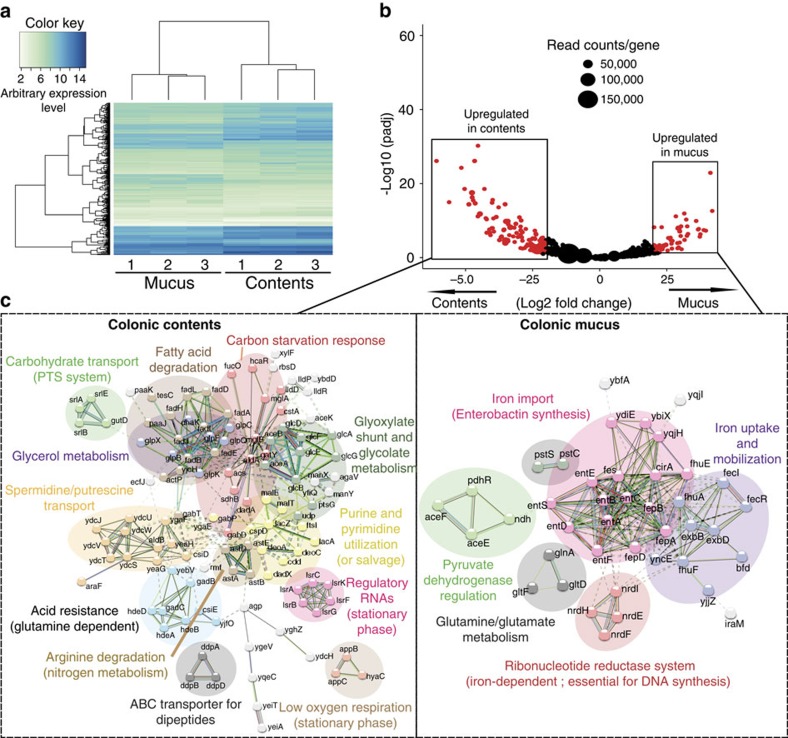
*E. coli* in colonic mucus and contents has different transcriptional patterns. (**a**) A heat map of gene expression values shows differentially expressed genes identified by RNA-seq in bacteria isolated from colonic mucus and contents of *E. coli* monocolonized C57BL/6 mice. Data include three experimental repeats. (**b**) A volcano plot was constructed as in Fig. 2b visualizing *E. coli* transcriptional patterns. (**c**) Network analysis on *E. coli* was performed as in Fig. 2c. Original transcriptomic data with annotations are given in Supplementary Data 2.

We first carried out transcriptomic analyses of parallel luminal or outer mucus isolates. In both *B. thetaiotaomicron* (Fig. 2) and *E. coli* monocolonized mice (Fig. 3), we observed distinct transcriptional patterns in unsupervized analyses of replicate samples from the mucus, that differed from the transcriptional patterns of the same bacteria in the luminal contents (see also Supplementary Data 1 and 2 for further details). To determine the specifics of the niche transcriptional patterns, we applied a network analysis using STRING^25^. We improved the transcript annotation for *B. thetaoiotaomicron* available in STRING by using the SEED tools. The annotations also include the latest studies regarding the glycan utilization pathways in *B. thetatiotaomicron*^26^,^27^. For both of the model bacteria studied under monocolonization conditions, there were distinct metabolic pathway clusters depending on whether the transcriptome had been derived from the outer layer of mucus or the intestinal contents. For example, *B. thetaiotaomicron* had increased N-linked glycosidase transcription in the mucus compared with the intestinal contents (Fig. 2c), compatible with digestion of glycan side chains of mucus in this compartment and from shed dead host cells. However, luminal *B. thetaiotaomicron* expressed a distinct repertoire of glycoside hydrolases consistent with metabolism of dietary starch, mannans, arabinogalactans, xylose and galactose (Fig. 2c and Supplementary Data 3). We concluded that this transcriptional evidence was consistent with *B. thetaiotaomicron* selectively utilizing particular members of its rich repertoire of glycoside hydrolases to degrade host complex carbohydrates in the outer layer of mucus or complex dietary carbohydrates resistant to host digestion in the luminal intestinal contents. We also found relatively increased expression of capsular polysaccharide synthesis loci in *B. thetaiotaomicron* residing in the intestinal lumen. This may be a result of increased biofilm formation on particulate luminal material, such as undigested plant wall fragments as previously suggested from chemostat transcriptional profile studies^28^.

### Iron harvesting dictates *E. coli* transcriptional regulation

Given that the abundant glycoside hydrolase repertoire of *B. thetaiotaomicron* allows it to exploit a wide range of host-secreted or dietary-derived complex carbohydrates, depending on its exact niche in the intestine, we next asked how *E. coli* as a facultative anaerobe with its limited glycoside hydrolase repertoire (Supplementary Fig. 8) would adapt to life in these niches. We carried out comparative STRING analyses on the transcriptomes from the mucus or the large intestinal contents of *E. coli* monocolonized C57BL/6 mice that had been fed an identical diet used in *B. thetaiotaomicron* experiments. The predominant difference between *E. coli* residing in the mucus and in the intestinal contents was a notably increased expression of the ferric iron uptake pathway (Fig. 3c and Supplementary Data 3). In support of these transcriptional data for critical iron harvesting in the mucus, we found lower total iron levels in colonic mucus (26.99±22.16 ng g^−1^, 
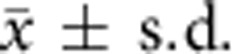
, *n*=3 mice) compared with the luminal contents (120.64±35.07 ng g^−1^).

**Figure 4:**
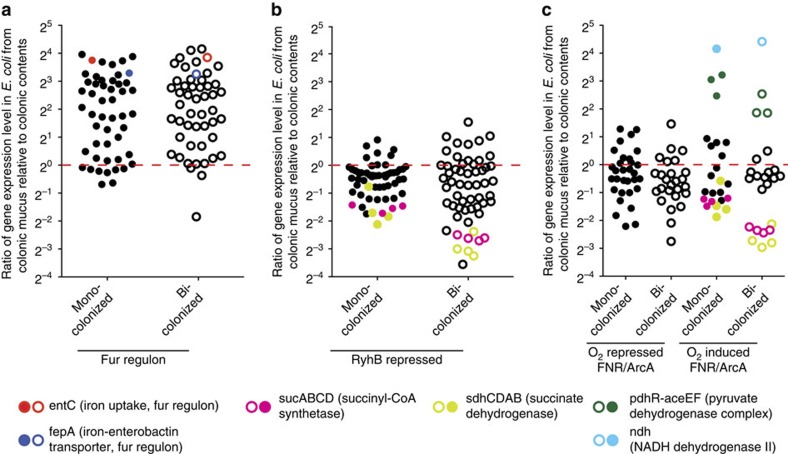
Iron recovery predominates behaviours of *E. coli* in colonic mucus. Genes (**a**) belonging to Fur regulon^29^ or (**b**) repressed by RyhB^30^ or (**c**) responding to oxygen levels (ArcA/FNR)^31^ of *E. coli* as reported previously were listed out. The ratio of expression levels of the whole panel of genes in colonic mucus relative to colonic contents is shown. Several genes/clusters were highlighted in indicated colours referring to same genes shown in Supplementary Fig. 9. The annotations of all displayed dots were listed in Supplementary Data 4. The red dotted line indicates a ratio of unity between colonic mucus and contents.

Fe^3+^ uptake genes are negatively regulated through the Fe-bound Fur master regulator^29^, with counter-regulation of iron-storage genes mediated through Fe-Fur repression of RyhB^30^. Expression ratios of members of the Fur/RyhB regulon showed that the system dominated the *E. coli in vivo* transcriptional repertoire in the colonic mucus in both *E. coli* monocolonized mice and when *B. thetaiotaomicron* was present in bicolonized mice (Fig. 4a,b and Supplementary Data 4). In contrast, there was a very modest effect of oxygen-driven differences between the two compartments of the known oxygen-sensitive genes in *E. coli*^31^, most were equally expressed in the mucus relative to the intestinal contents, with only NADH dehydrogenase II complex and pyruvate dehydrogenase upregulated in the mucus (Fig. 4c and Supplementary Data 4). These *E. coli* transcriptomic signatures were also found in mice colonized with both *E. coli* and *B. thetaiotaomicron* (Fig. 4 and Supplementary Data 4). Succinate dehydrogenase and succinyl CoA synthase, which are induced by oxygen and repressed by Fur/RyhB^32^, showed decreased mucus/contents expression ratios in line with the predominant requirement for iron harvesting by mucus bacteria (Fig. 4b,c).

**Figure 5:**
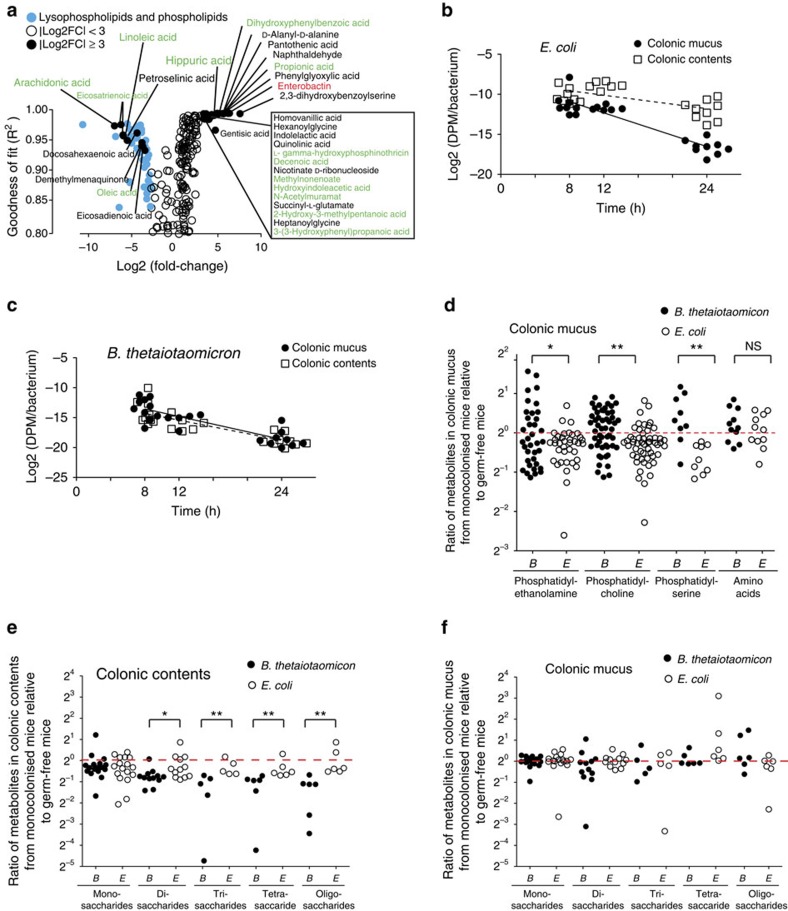
*Ex vivo* and *in vivo* metabolites consumption assay and bacterial replication dynamics. (**a**) *E. coli* was cultured on colonic mucus and luminal contents harvested from germ-free mice (*n*=4). The consumed and secreted metabolites were identified at sequential time points comparing metabolic patterns before and after *E. coli* growth by non-targeted mass spectrometry analysis. *R*^2^ values representing the goodness of exponential fit of nonlinear regression indicate the continuity of metabolite variation trend among the time course. Metabolites with annotations labelled in green indicate possible alternative molecular species desigations (listed in full in Supplementary Data 5). (**b**) *E. coli* and (**c**) *B. thetaiotaomicron* replication rates in colonic mucus and contents from monocolonized mice were addressed by ^32^P-radioactivity decay after metabolic labelling of the test bacteria. Data are pooled from three independent experiments. (**d**) The average intensity of classified phospholipids detected directly *ex vivo* from colonic mucus from *E. coli* monocolonized mice (*n*=4), *B. thetaiotaomicron* monocolonized mice (*n*=4) were compared with germ-free mice (*n*=4). Comparison of detected common amino acids served as a control group. The red dotted line indicates ratios of unity between monocolonized and germ-free status. (**e**,**f**) Metabolites classified as oligosaccharides from (**e**) colonic contents or (**f**) colonic mucus were compared directly *ex vivo* as described for **d**. NS, not significant.

To confirm the transcriptomic data, we carried out metabolomic analyses of *ex vivo* cultures of *E. coli* on mucus harvested from germ-free mice. These showed markedly increased production of enterobactin—a very high-affinity iron-chelating molecule produced as a siderophore by *E. coli* to capture iron under limiting conditions of bioavailability^33^ (Fig. 5a and Supplementary Data 5). In contrast, *B. thetaiotaomicron*, which assimilates iron in the form of haem, showed no overall difference in expression levels of genes encoding haem uptake pathways (Supplementary Fig. 9a and Supplementary Data 4). Mammals use a variety of mechanisms to sequester ionic iron and limit its bioavailability in central body tissues: this generally limits the infectivity of microbes that need to assimilate iron in its ionic form^33^. Our data show that restriction of ionic iron also applies to the outer mucus layer of the colon and is consistent with the large number of iron metabolism genes found in intestinal bacteria compared with other host-associated niches (Supplementary Fig. 10).

### Non-preferred carbon source regulation in colonic contents

Transcriptional activity of luminal *E. coli* in monocolonized mice also showed evidence of a shift to non-preferred carbon sources including glycerol and fatty acid metabolism (FAD operon)^34^, with additional upregulation of transcript pathways annotated as the carbon starvation response and glyoxylate shunt (Fig. 3c). Regulatory RNAs associated with the transition to stationary phase were also upregulated. This is indirect evidence that replication of *E. coli* within the intestinal lumen was restricted by the limited availability of suitable metabolizable carbon source. An overall shift to non-preferred carbon sources was also documented by an overall increased luminal expression of genes positively regulated by the cAMP-CRP catabolite (de)repression system^35^ (Supplementary Fig. 9b and Supplementary Data 4). As with oxygen-sensitive gene regulation, the effect of the Fur/RyhB system dominated mucus expression of those genes that would otherwise be repressed by CRP-cAMP (Fig. 4a and Supplementary Fig. 9b). These data collectively confirm that *in vivo* control of iron harvesting in mucus by *E. coli* takes precedence over carbon source preference or oxygen availability.

### Mucus/luminal differential bacterial proliferation rates

Large intestinal transit measurements are variable, depending on technique, diet, stress and the amount of physical activity, but are generally measured in mice in the range of 1–5 h (ref. 36), so microbiotas need to be highly regenerative, either from proximal reinoculation or through local proliferation to avoid washout. We next carried out direct measurements to confirm our interpretations from bacterial morphology (Supplementary Fig. 6e and f) and transcriptomics (Fig. 3c) that *E. coli* is replicating quickly in the outer layer of mucus but enters stationary phase in the intestinal contents. This was also predicted indirectly in earlier studies of regrowth of *E. coli* in streptomycin-treated mice, either from bacterial ribosomal content measurements^37^ or from impaired colonization with mutants having reduced potential for mucus-supported growth^10^. We measured bacterial proliferation directly using either flow cytometry to determine the sequential mean fluorescent intensity reduction per bacterium following each division in an *E. coli* strain where green-fluorescence protein had been pre-induced 38 (Supplementary Fig. 11) or through loss of specific radioactivity in *E. coli* or *B. thetaiotaomicron* that had been metabolically labelled by growth in ^32^P-containing medium (Fig. 5b,c and Supplementary Table 1). Both methods showed that the *E. coli* in the outer mucus layer proliferated with an estimated half-time of 3 h (whether studied during monocolonization or in the presence of *B. thetaiotaomicron*; Supplementary Table 1), whereas *E. coli* proliferation in the intestinal contents was far slower with an estimated half-time of 8 h (Fig. 5b). We predicted that *B. thetaiotaomicron* with its extended glycoside hydrolase metabolic capacity should be able to replicate equally well in both compartments. This was confirmed by following the trajectory of specific radioactivity loss in the bacteria inhabiting either the outer mucus layer or the intestinal contents: in both compartments, *B. thetaiotaomicron* replicated with an estimated division time of 3 h (Fig. 5c). Given its rather restricted replicative niche, it is significant that *E. coli* makes only a small contribution to the overall intestinal microbial numbers of a healthy host, whereas *B. thetaiotaomicron,* which replicates in both mucus and luminal niches, is relatively abundant. Conversely, under conditions of infectious^21^ or non-infectious inflammation^22^, or even following diagnostic intestinal purging^39^, when the luminal intestinal carbon biomass is decreased, there is a relative bloom of Gammaproteobacteria and relative decrease of Bacteroidetes.

### Host-derived phospholipids utilized by mucus *E. coli*

*E. coli* must sustain its replicative activity in the outer layer of the intestinal mucus despite a rather restricted ability to degrade complex carbohydrates. Given the high transcriptional signals of catabolite de-repression through the cAMP-CRP global regulatory system following shedding into the luminal contents (Supplementary Fig. 9b), we then asked whether *E. coli* was starting to use other non-preferred carbon sources even in the intestinal mucus. In mass spectrometric analysis of the intestinal mucus, we saw evidence of abundant presence of phospholipids: these included phosphatidylcholine (Supplementary Fig. 12a), which must be host derived, as it is not synthesized by *E. coli*^40^. Direct measurements of metabolite utilization from the cell-free medium when *E. coli* was cultured on germ-free mucus in an *ex vivo* culture system showed progressive consumption of over 42 different phospholipids (Fig. 5a and Supplementary Fig. 12b and Supplementary Data 5), suggesting that these (largely host-derived carbon sources) could be potentially exploited during growth in mucus. Neither such significant phospholipid consumption nor metabolomic signatures of iron salvage were detected during equivalent experiments where either *E. coli* was cultured in intestinal fluid (Supplementary Fig. 12c and Supplementary Data 6) or where *B. thetaiotaomicron* was grown on germ-free mucus (Supplementary Fig. 12d and Supplementary Data 7). The lack of *B. thetaiotaomicron* consumption of host phospholipids can be understood in terms of its genome-encoded metabolic repertoire, as it is one of the bacterial genera lacking the genes of the fatty acid (β-oxidation) operon (Supplementary Fig. 13).

To substantiate these phospholipid consumption data from the *ex vivo* culture system *in vivo*, we compared measurements of the steady-state levels of phospholipids in the cell-free mucus fractions of either *E. coli-*colonized or *B. thetaiotaomicron*-colonized mice with germ-free controls. These showed that there are lower steady-state levels of phosphatidylcholine, phosphatidlyethanolamine and phosphatidylserine in the dynamically regenerating mucus of *E. coli* monocolonized mice, but not in *B. thetaiotaomicron* monocolonized mice (Fig. 5d and Supplementary Data 8). Given that phosphatidylcholine is not synthesized by *E. coli*^40^, this is consistent with the consumption of host-derived phospholipids as a non-essential contributory carbon source in the outer mucus layer. In contrast, we found that *B. thetaiotaomicron* monocolonization, but not *E. coli* monocolonization, reduced oligosaccharides levels selectively in the intestinal luminal contents compared with germ-free mice (Fig. 5e,f and Supplementary Data 8 and 9). Whereas monosaccharide levels are equivalently very low for both organisms in the mucus and the intestinal contents, *E. coli* in contrast to *B. thetaiotaomicron* lacks the necessary metabolic pathways to harvest more complex oligosaccharides in the intestinal lumen: this is consistent with the carbon starvation transcriptional signature observed for *E. coli* in the luminal compartment (Fig. 3c).

### *E. coli* and *B. thetaiotaomicron* interactions during bicolonization

**Figure 6:**
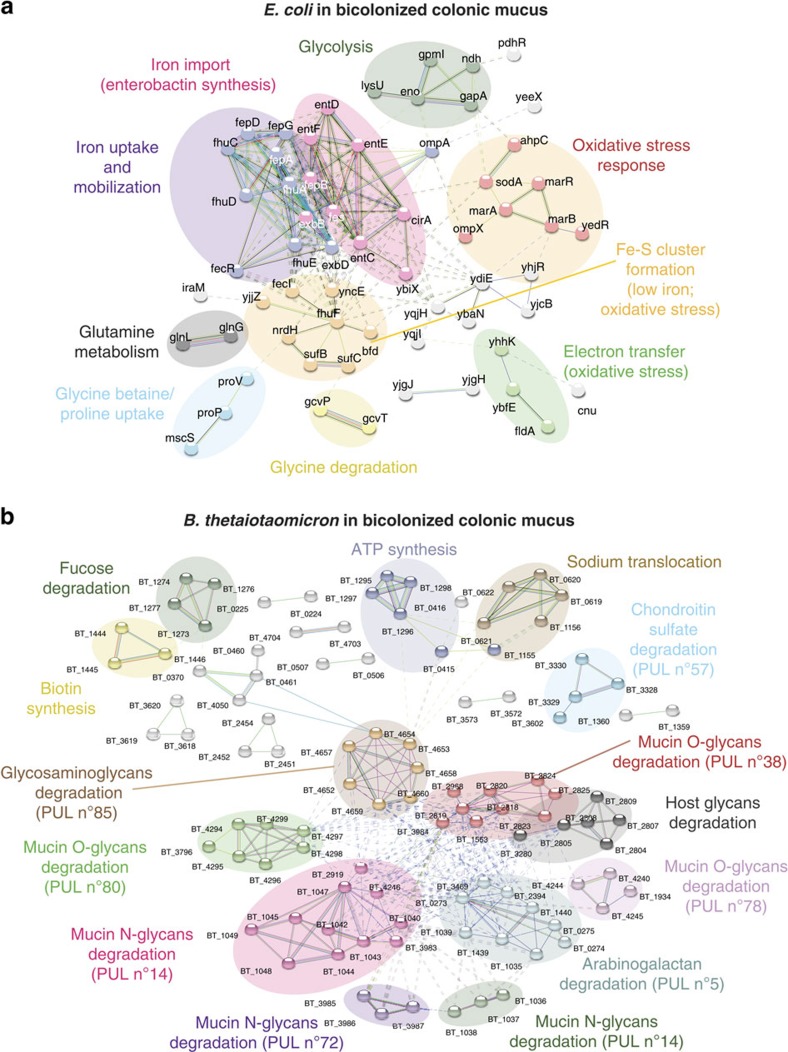
RNAseq and STRING analysis on *E. coli* and *B. thetaiotaomicron* from bicolonized mice mucus. Network analysis on transcriptional patterns of *E. coli* in colonic mucus (**a**) and *B. thetaiotaomicron* in colonic mucus (**b**) from *E. coli* and *B. thetaiotaomicron* bicolonized mice were performed as in Fig. 2c. Original transcriptomic data with annotations are given in Supplementary Data 10 and 11.

**Figure 7:**
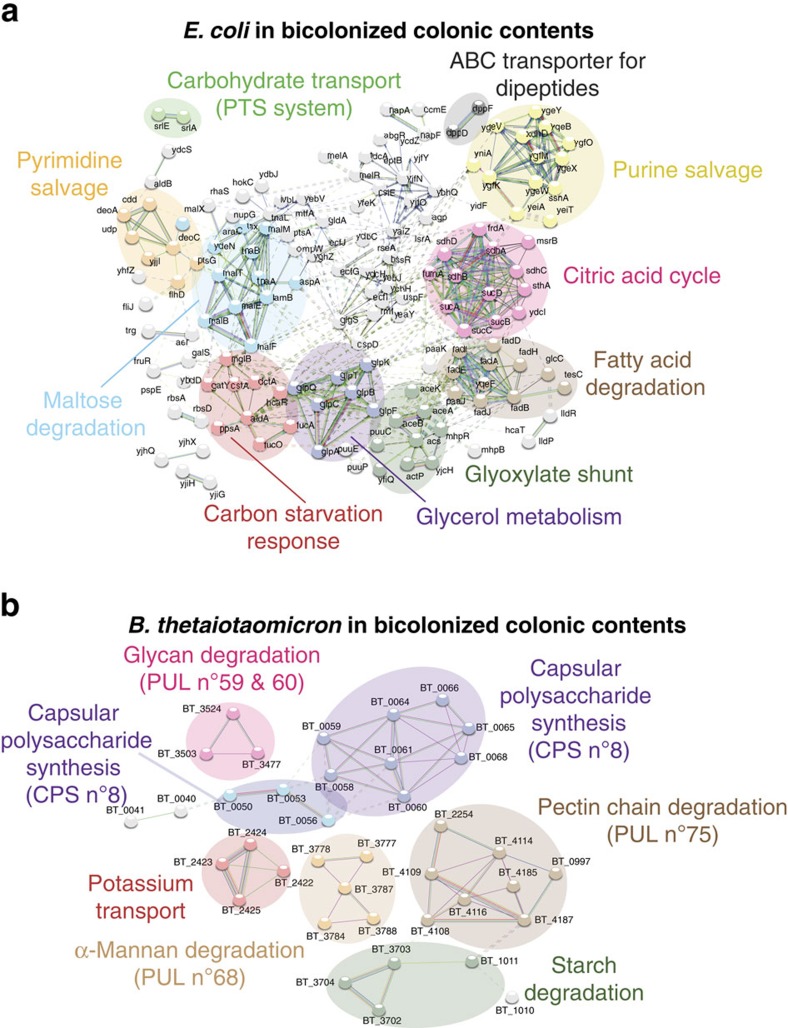
RNAseq and STRING analysis on *E. coli* and *B. thetaiotaomicron* from bicolonized mice contents. Network analysis on transcriptional patterns of *E. coli* in colonic contents (**a**) and *B. thetaiotaomicron* in colonic contents (**b**) from *E. coli* and *B. thetaiotaomicron* bicolonized mice were performed as in Fig. 2c. Original transcriptomic data with annotations are given in Supplementary Data 10 and 11.

We next asked how simultaneous colonization with *B. thetaiotaomicron* and *E. coli* could influence each other's metabolic gene expression and monitored niche-specific metatranscriptomic analysis of bicolonized mice. Our object here was to examine interactions between two microbial species having other distinct strategies for existence in different niches where we did not expect significant synergies or antagonism. The results of differential carbon source utilization, iron uptake and inferred replicative efficiency in both bacteria in the different niches of bicolonized mice were very similar to those of each individual in monocolonized mice (Figs 6 and 7, and Supplementary Data 10 and 11). Direct measurements of replicative efficiency showed that *E. coli* still only proliferated efficiently in the mucus, as in monocolonized mice (Supplementary Table 1). Individual analysis of bacterial gene expression driven by the different global regulators (CRP, Fur/RhyB, FNR/ArcA) in the bicolonized mice also showed results consistent with the stratification of mineral uptake and carbon source preference seen in monocolonized mice (Fig. 4 and Supplementary Fig. 9b). Bicolonization did cause an increase the range of polysaccharide utilization loci of *B. thetaiotaomicron* with additional access to mucin O-glycans in the colonic mucus presumably as a result of increased glycan exposure (compare Fig. 2c and Fig. 6b). Genes for maltose utilization in *E. coli* were relatively increased in the intestinal contents of bicolonized mice, presumably as a consequence of cross-feeding (Fig. 7a). There were also transcriptional signals of increased oxidative stress in the mucus niche for both microbes (Fig. 6a, Supplementary Fig. 14a and b and Supplementary Data 4). For *E. coli,* these particularly affected the superoxide dismutase (*sodA*) and the *arc*, *ahp*, *mar* and *suf* operons. For *B. thetaiotaomicron* in the mucus of bicolonized mice, catalase (BT_1971), alkylhydroperoxide reductase (BT_2011 and BT_2012), thiol peroxidase (BT_1329) and reverse rubrerythrin (BT_0216) were induced. Genes activated by the *E. coli* sigma-S global stress regulator^41^ generally showed increased expression in mucus of bicolonized mice (Supplementary Fig. 14c and Supplementary Data 4). Oxidative stress effects are likely due to reactive oxygen species generated as a result of (bacterial) contact-independent small-molecule membrane perturbation or cell wall damage^42^.

**Figure 8:**
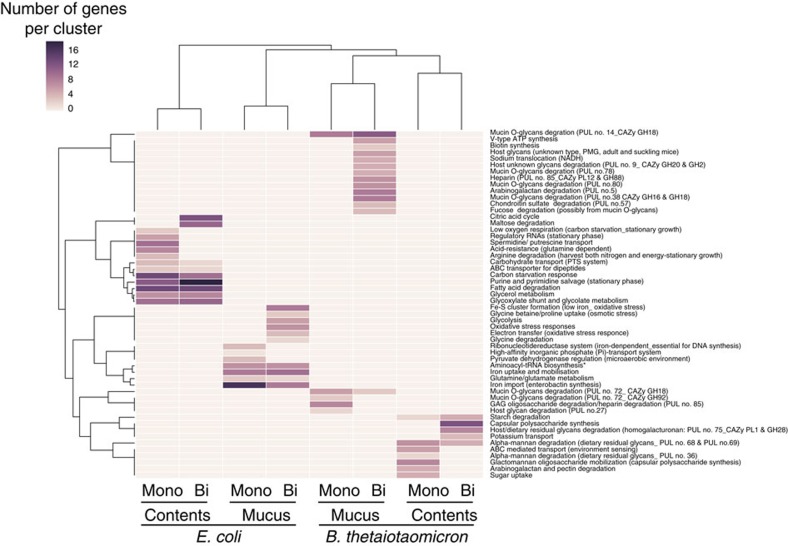
Gene cluster distribution allows discrimination between mucus and content compartments. A heat map of the correlation of each cluster identified in the STRING analysis and the number of genes each cluster is shown. The unsupervised profiles for each experimental condition clustered *E. coli* and *B. thetaiotaomicron* separately, whereas regrouping profiles for monocolonization and bicolonization for each compartment and microbe.

A heat map of the correlation of each cluster identified in the STRING analysis and the number of genes per cluster discriminates the gene cluster distribution between both species and both niches (Fig. 8). The obtained profiles for each experimental condition regroup profiles for bicolonized and monocolonized conditions confirming the robustness of expression profiles despite the environmental change. Thus, the cluster distribution shows specific expressions upon bicolonization, for example, in *B. thetaiotaomicron* (Fig. 8), which indirectly reflect a switch in the diversity of polysaccharide utilization.

## Discussion

The stratification between the outer layer of mucus and the luminal contents is important from the standpoint of host microbial mutualism. Diversely colonized mice and medium diversity gnotobiotic mice have different representations of microbes in the outer mucus layer compared with the luminal intestinal contents, even though luminal contents are inevitably contaminated by biomass shed from the mucus. Although most bacterial species inhabit both compartments, there is a special niche in the mucus with differential resource utilization and particular strategies for population compared with the same species in the intestinal lumen. Our illustration of stratification of 'fundamental' niches is a starting point to investigate the real-life complexity of 'realized' niches in which competition and synergies between different members of the microbial communities shapes their overall structure and function^11^,^13^,^43^. These realized niches will also be influenced by the increased thickness of colonic mucus in the presence of complex microbiotas and pathobionts, and by variability in the host diet.

There are a number of reasons to consider that microbes in the outer mucus layer may have a special functional role in the host–microbial relationship. First, although inner mucus separates the microbes themselves from the epithelial layer, molecular exchange occurs bidirectionally between the outer mucus layer and the host, and there is also molecular uptake into the host through goblet cells during the secretory process^44^,^45^. Many microbial molecules can enter the host^46^, including short-chain fatty acids that feed host epithelial cells^47^ and regulate immunity^48^,^49^. Molecular uptake from microbes is presumably most potent when they are adjacent to host tissues. Second, the mucus barrier protects against mucosal infections^50^; this effect is complemented in the intestine by the endogenous microbiota that fills this microbial niche in which enteric pathogens and opportunistic pathobionts must face intense competition to initiate infections. Third, some bacterial mutants that are unable to metabolize mucus constituents are poor colonizers compared with their parent strain^51^, so adaptation to life in this layer may determine survival or extinction in the host. Finally, the mucus layer is a very challenging habitat as it is undergoing rapid renewal with a timescale of several hours. This means that microbes must be fit enough to be renewed or replenished at the same rate while competing with each other for resources to persist within this especially intimate and stressful niche of the host–microbial intestinal biomass.

Much of the current literature, which seeks to relate intestinal microbiotas to host phenotypes or diet, is dominated by composition assessments of the microbes and their genomic composition in the luminal contents or in the excreta of human and animal hosts. Although such assessments can provide a first approximation of the microbiota biomass and metabolic potential, they do not capture actual metabolic variations along the length of the intestine or—as we now show—the different transverse niches. The colonic mucus niche harbours microbes that adapt their metabolism to the availability of shed host compounds, according to their genome-encoded repertoire. These functional differences, presented from studies in gnotobiotic mice, provide an insight into the likely complexity of metabolic interactions, both within consortia, and between consortia and their host. The functional interactions within microbial members of natural complex microbiota and their host are likely to vary not only according to community composition and diet, but also with position transversely and longitudinally in the intestinal tract.

## Methods

### Mice and hygiene status

C57BL/6 mice were re-derived to germ-free status^52^. Gnotobiotic C57BL/6 mice colonized with ASF or sDMDMm_2_ containing 12 defined bacterial strains (called the Oligo-Mouse-Microbiota, see ‘Bacterial strains') were generated and maintained at the clean mouse facility of the University of Bern. SPF mice (on a C57BL/6 background) were purchased from Harlan Laboratories. Monocolonization was performed by intragastric administration of 10^10^ colony-forming units (CFU; if not otherwise specified) of *E. coli* or *B. thetaiotaomicron* into 8- to 16-week-old mixed-sex germ-free mice. Bicolonization was established in 8- to 16-week-old mixed-sex germ-free mice by orally gavaging with 10^10^ CFU of *E. coli* and 10^10^ CFU of *B. thetaiotaomicron*. Mice were either used germ-free or stably colonized over 5 days with the organism(s) shown, except for trajectory experiments for bacterial replication where time points between 8 and 24 h were used. Control experiments confirmed that the dose of 10^10^ CFU was saturating and established a steady state at the outset, whereas doses ≤10^8^ CFU required an initial phase of replication before the biomass reached steady state at 18 h. Germ-free mice were routinely monitored by culture-dependent (Luria-Bertani Broth (LB) agar and Wilkin's Anaerobic media (Oxoid) supplemented with 5% defibrinated sheep's blood) and culture-independent (Gram stain and cell-impermeant nucleic acid stain Sytox green) methods to confirm sterility. All mouse experiments were performed in accordance with the Swiss Federal and Cantonal regulations and were approved by the Ethics Committee for Animal Experimentation of the SAMS and SCNAT.

### Bacterial strains

*Escherichia coli* strains MG1655 (ATCC70092) and JM83 (ATCC35607) were purchased from American Type Culture Collection (ATCC) and maintained in lab and used as specified. The immotile *ΔflhDC* mutant of MG1655 and wild-type motile strains was kindly provided by Dr Paul Cohen^53^. *B. thetaiotaomicron* VPI-5482 (ATCC no. 29148) were grown in Schaedler's broth anaerobically. The Oligo-Mouse-Microbiota includes *Bacteroides I48, Blautia YL58, Akkermansia YL44, Bacteroidales YL27, Ruminococcaceae KB18, Lactobacillus I49, Lachnospiraceae YL32, Erysipelotrichaceae I46, Enterococcus KB1, Flavonifractor YL31, Parasutterella YL45* and *Bifidobacterium YL2* (detailed microbiological characterization will be separately reported, strain requests to stecher@mvp.uni-muenchen.de).

### Plasmids

Plasmid pDIGc contains a constitutive GFP expression element, whereas plasmid pDIGi was constructed with an isopropyl-β-D-thiogalactoside-inducible GFP expression element^38^. Both of these plasmids confer ampicillin resistance and were transformed into bacteria by electroporation using Gene Pulser Xcell (Bio-Rad).

### Immunofluorescence

Colon segments with contents maintained were fixed in Methanol–Carnoy's fixative according to a published protocol^54^ with a few modifications. After 4 h of fixation in Methanol–Carnoy's, samples were incubated for 2 × 30 min in methanol, 2 × 20 min in ethanol, 2 × 25 min in xylene and 2 × 30 min in liquid paraffin before paraffin embedding.

To visualize intestinal mucus, 6 μm colon sections were stained for 1 h with polyclonal rabbit anti-mouse Muc2 (1:50, sc-15334, Santa Cruz), and mouse serum (1:50) collected 21 days after systemic priming with *E. coli*. After three washes in histological buffer (0.9 M NaCl, 20 mM Tris-HCl, pH 7.2), sections were incubated with DyLight549-conjugated goat anti-rabbit IgG (1:100, 111-505-144, Jackson Immunoresearch), FITC-conjugated rat anti-mouse IgG2b (1:100, 553395, BD) and 0.5 μg per ml DAPI (4′,6′-diamidino-2-phenylindole) and imaged using Nikon Eclipse 800 fluorescence microscope.

### Mucus thickness measurement

The *ex vivo* mucus measurement was performed according to a previous publication^55^ with a few modifications. Germ-free mice were colonized with indicated bacteria (*E. coli* and *B. thetaiotaomicron*) over 5 days, or born and raised at germ-free, ASF or SPF hygiene conditions for over 30 generations. Mice were intravenously injected with 100 μl Evan's blue (20 mg ml^−1^, E2129, Sigma) to visualize the epithelial layer. After 3 min, segments of the intestine were removed and opened longitudinally. The luminal contents were gently removed and then 0.5% charcoal/PBS was sprinkled over the apical surface to visualize the top of the mucus layer. Using a microdispenser connected to a micromanipulator (Drummond Scientific) with a glass capillary (>0.05 mm tip diameter), the distance between the charcoal layer and the epithelium was measured in five different spots within the section (that is, colon) per mouse. To determine the inner mucus thickness, the outer layer was aspirated before measurements. Mucus thickness was then calculated using the formula: Sin(angle of sample sheet to glass capillary) × measured distance=mucus thickness.

### Collection of mucus and luminal contents

Intestinal segments were taken from mice and opened longitudinally. The contents were gently removed using forceps without scraping the surface. The remaining contents on the colonic tissue sheet were picked away until no visible particles remained. A pipette tip was linked to the soft tube of a vacuum pump. Using gentle vacuum, the mucus was sucked into the tip. By pipetting, mucus was transferred to Eppendorf tubes containing 100 μl PBS.

### Microbial community analysis

The 16S rRNA gene segments spanning the variable V5 and V6 regions were amplified from DNA from mucus, contents or faecal samples using a multiplex approach with the barcoded forward fusion primer 5′-*CCATCTCATCCCTGCGTGTCTCCGACTCAG* BARCODE ATTAGATACCCYGGTAGTCC-3′ in combination with the reverse fusion primer 5′-*CCTCTCTATGGGCAGTCGGTGAT*ACG AGCTGACGACARCCATG-3′. The sequences in *italics* are Ion torrent PGM-specific adaptor sequences. The PCR amplified 16S V5-V6 amplicons were purified and prepared for sequencing on the Ion torrent PGM system according to the manufacturer's instructions (Life Technologies). Samples with over 500 reads were accepted for analysis. Data analysis was performed using the QIIME pipeline version 1.8.0. Operational taxonomic units were picked using UCLUST^55^ with a 97% sequence identity threshold followed by taxonomy assignment using either the latest Greengenes database (http://greengenes.secondgenome.com) for SPF samples or a custom sDMDMm_2_ database (Supplementary Data 12) for gnotobiotic sDMDMm_2_ samples. Calculation of the Shannon index, weighted and unweighted UniFrac-based PCoA, and statistical analysis using Adonis were all performed using the QIIME pipeline^56^.

### Two-photon microscopy

Colon sections from germ-free mice monocolonized with *E. coli* MG1655-pDIGc were examined immediately *ex vivo*. The luminal contents were gently removed with forceps and the tissue was stained with 1 μg ml^−1^ Hoechst blue (62249, Thermo Scientific) for 1 min on basolateral surface, and then glued onto microscopy slides. After immersing the sample in water, two-photon laser scanning was performed using a × 20 objective (numerical aperture=0.95, Olympus) on a TrimScope microscope platform (LaVision Biotec). A Ti:sapphire laser (Mai Tai HP) set to 800 nm as the excitation wavelength, and 447/55 nm, 525/50 nm, 593/40 nm and 655/40 nm bandpass filters were used to acquire emitted light and second harmonic signals for generating colour images. Bacterial tumbling was traced on one fixed *x*–*y* section (300 × 300) around 50 μm above the epithelial surface with 1s time intervals for 5 min. Sequences of images were transformed into time-lapse videos with Volocity software (PerkinElmer).

### Mucus turnover

Incorporation of *N*-acetylgalactosamine (ARC0103, American Radiolabeled Chemicals) to mucus was measured during Muc2 biosynthesis through O-linked glycosylation of radiolabel, and pulse-chasing GalNAc labelling of mucus was used to assess the mucus turnover^14^. [^14^C]-GalNAc (10 μCi) was diluted in 500 μl PBS and i.p. injected into ASF colonized mice. Mucus and luminal contents were collected from the colon up to 32 h and counted for radioactivity. The mucus turnover time after the lag time for the appearance of newly synthesized mucus was calculated as DPM per gram^max at t=11∼12h^ – DPM per gram^min at t=8h^.

### Bacterial RNA-seq

Pooled colonic mucus or luminal contents (*n*=3 per group of *E. coli* JM83 monocolonized mice, *n*=4 per group of *B. thetaiotaomicron* monocolonized mice and *n*=3 per group of *E. coli* JM83 and *B. thetaiotaomicron* bicolonized mice) were collected and homogenized in 0.5% Tergitol/PBS containing 5% RNase inhibitor (RNaseOUT, Invitrogen). After a quick spin at 200*g*, the remaining supernatant was transferred to a new tube and centrifuged for 10 min at 6,000*g* to pellet bacterial-sized particles. The bacterial portion in the pellets was enriched by centrifugation for 30 min at 670*g* on a 50%/80% Percoll gradients (17-0891-01, GE Health) for *E. coli* monocolonized, 40%/70% for *B. thetaiotaomicron* monocolonized or 40%/80% for bicolonized samples. The bacterial layer collected from the interface was lysed in 100 μl RNA extraction buffer^57^, treated with DNase (RNase-Free DNase Set, Qiagen), and purified using RNeasy clean-up kit (Invitrogen). The RNA concentration was measured using Bioanalyzer 2100 (Agilent). rRNA was depleted (Ribominus Transcriptome Isolation Kit, Invitrogen) and the RNA was concentrated (RNA Clean&Concentrator, Zymo Research) for cDNA synthesis and Library preparation (TruSeq Stranded mRNA Sample Preparation, Illumina). Libraries were sequenced by Illumina HiSeq 2500 with 125bp paired-end mode. Reads were mapped to the reference genomes (*E. coli*: strain K-12 substrain MG1655 (Assembly GCA_000005845.1.20); *B. thetaiotaomicron*: strain VPI-5482 (Assembly GCA_000011065.1) using Bowtie2 v.2.2.1 (ref. 58), and the number of reads overlapping annotated genes was counted with HTSeq v. 0.6.1 (ref. 59). DESeq2 (ref. 60) was used to test for differential expression between groups.

### Network analysis for the differentially expressed genes

For each RNA-seq data set, genes were ranked for their absolute log2FoldChange ≥ 2 comparing the gene expression level in mucus to luminal contents or vice versa and adjusted *P*-value of <0.05. For *E. coli* in monocolonized mice, the mucus high-expressed and luminal contents' high-expressed gene sets contained, respectively, 52 and 121 genes. For *E. coli* in bicolonized mice, the genes were 81 in mucus and 177 in luminal content. For *B. thetaiotaomicron* in monocolonized mice, the mucus and luminal contents' gene sets contained, respectively, 35 and 50 genes. For *B. thetaiotaomicron* in bicolonized mice, the gene counts were 50 in mucus and 118 in luminal content. The proteins corresponding to the obtained gene sets were searched against the STRING database version 9.1(ref. 61) for protein–protein interactions. Although a recent paper describes the version 10 of the STRING database^62^, this update was not available online during analysis, therefore all the work in this study was performed using version 9.1 with appropriate customization (J.P.L) to ensure currency of the database. The STRING software compiles available experimental evidence for the reconstruction of functional protein-association networks. The respective bacterial protein interactions data set was selected for each previously described gene set. For each interaction, STRING specifies a metric called ‘confidence score'. All interactions fetched in our analysis had a confidence score ≥ 0.4 (medium+high confidence). Additional information for the *B. thetaiotaomicron* interactomes based on our curated genome annotation is available via a payload version of STRING-DB at the address http://string-db.org/newstring_cgi/show_input_page.pl?external_ payload_URL=http://string.mucosalimmunology.ch/Btheta_payload.json.

### Clustering and function enrichment analysis

The interaction networks were analysed for densely connected regions using the embedded clustering methods in STRING. Multiple rounds of iteration of the Kmeans clustering method using different k seed numbers were performed. The obtained clusters were tested for their significance using the analysis tool in STRING and tested for enrichment for KEGG pathways. Because of the classification of certain genes in multiple KEGG pathways we performed a manual curation of those using extensive literature and database search to refine the functional description of the Kmean clusters.

### Reannotation of *Bacteroides thetaiotaomicron's* genome

The genome annotation of *B. thetaiotaomicron* present in the version 9.1 of the STRINGDB still largely referred to the original annotation from 2003. We therefore performed a re-annotation of the genome using the automated RAST-SEED platform to update the annotation according to the most recent methods and databases^63^,^64^. We recovered the new annotation data for the genes differentially expressed in our data sets and used it as a scaffold to integrate *B. thetaiotaomicron*-specific information we retrieved from an extensive screening of the literature and databases (for example, CAZY.org). This work was performed to implement the most recent biochemical characterization of *B. thetaiotaomicron*.

### Data mining of the Human metabolome database and data visualization

The bacterial genome data from the Human microbiome project (HMP) were retrieved from the Human microbiome project reference genomes data repository (http://www.hmpdacc/HMRGD). The data were analysed using custom python scripts and the integrated microbial genomes database and comparative analysis system^65^. The information for the phylogenetic distribution of the four core enzymes implicated in the β-oxidation (E.C. 4.2.1.17, 1.1.1.35, 2.3.1.16, 2.3.1.9) was retrieved for 138 bacterial genomes representatives of the main genera of the bacterial tree. The data were visualized on the interactive Tree Of Life iTOL^66^. The occurrence of enzymes implicated in iron metabolism, as predicted by the automated annotation used by the HMP, was inferred for the 1,317 genomes of the HMP reference genomes (as of January 2015).

### Single-cell bacterial replication dynamics

*E. coli* MG1655 carrying pDIGi was cultured overnight with 0.5 mg ml^−1^ ampicillin and 0.5 mM isopropyl-β-D-thiogalactoside to induce GFP expression. Bacteria were cultured in LB broth without supplements to check the bacterial replication dynamics correlated to GFP dilution. For *in vivo* assays, 10^10^ GFP-positive *E. coli* MG1655 was intragastrically administered to germ-free mice and loss of GFP signal was assessed by flow cytometry at different time points following gavage. The samples were collected from mice and homogenized in 0.5% Tergitol/PBS. A filtration using 40 μm nylon filter was done on samples before flow cytometric acquisition. After sample acquisition on a FACSArray (BD), the results are analysed by Flowjo (Version 9.5.3).

### Bacterial *in vivo* replication assay by radioactivity

Bacteria were radiolabelled by supplementing [^32^P] phosphate (NEX011002MC, PerkinElmer), to cultures at 5 μCi per 30 ml for each mouse for *E. coli* and 10 μCi per 50 ml for each mouse for *B. thetaiotaomicron*. After oral gavage of 10^10^ CFU [^32^P] bacteria to germ-free mice, the colonic mucus and contents were isolated at 8, 12 and 24 h after gavage. Bacterial CFU was determined by culture of half of each sample on LB plates (*E. coli)* or blood agar plates (*B. thetaiotaomicron*). The other half of each sample was homogenized in 1 ml of NCS II Tissue solubilizer (GE Healthcare) for a minimum of 1 h at 56 °C. Once solubilized, 100 μl of glacial acetic acid and 18 ml of ULTIMA Gold liquid scintillation cocktail (PerkinElmer) was added and the level of ^32^P radioactivity was measured in disintegrations per minute (DPM) using a TRI-Carb 2300TR Liquid Scintillation Analyzer (Packard). Colorimetric quench curves were created using caecal contents to ensure accurate measuring of ^32^P in biological samples. Baseline levels of ^32^P were determined by measuring the levels of radioactivity in germ-free mice. The replication rate of bacteria was determined by calculating 1 slope^−1^ of linear regression of DPM bacterium^−1^.

### *Ex vivo* and *in vivo* metabolic consumption assay

For the *ex vivo* consumption assay, colonic mucus and contents were collected from germ-free mice and homogenized in M9 minimal media. After removal of particulate material from the homogenate, the supernatants were transferred to 96-deep-well bacterial culture plates. 10^4^ CFU of *E. coli* or *B. thetaiotaomicron* (*B. thetaiotaomicron* cultures were also supplemented with 1 μM vitamin B6 and 5.8 μM vitamin K3) were inoculated into each well and cultured at 37 °C anaerobically. Aliquots of the culture were collected at 0, 1, 2, 3, 6, 9 and 24 h. Bacteria were removed by centrifugation and the supernatants were subjected to mass spectrometry (MS) after precipitation of the MS undetectable large molecules by adding four volumes of methanol.

For the *in vivo* metabolites assay, 10^10^ CFU *E. coli* or *B. thetaiotaomicron* were gavaged into germ-free mice. Colonic mucus and contents were collected from monocolonized mice and control germ-free mice. Hot water extraction was performed on samples by continuous heavy shaking at 80 °C for 3 min. Particles were pelleted by centrifugation and supernatants were subjected to analysis on MS.

A 6550 Agilent Q-TOF mass spectrometer was used for measuring metabolites by untargeted flow injection analysis as described previously^67^. Profile spectra with high mass accuracy were recorded from 50 to 1,000 m/z in negative ionization mode. Ions were annotated based on accurate mass comparison using 5 mDa mass tolerance against 9,261 unique metabolites present in the Human Metabolome Database^68^.

### Statistics

Graphpad Prism 6 software was used for both statistical analysis and to perform linear and nonlinear regression analysis. Statistical tests used were Mann–Whitney test (*P* < 0.05) and paired/unpaired Student's *t*-test. Adonis statistics were performed using QIIME^56^.

## Additional information

**Accession codes**: The 16S sequence data have been deposited in Figshare and can be accessed through the following link: http://dx.doi.org/10.6084/m9.figshare.1499145.

**How to cite this article:** Li, H. *et al.* The outer mucus layer hosts a distinct intestinal microbial niche. *Nat. Commun.* 6:8292 doi: 10.1038/ncomms9292 (2015).

## Supplementary Material

Supplementary Figures and Supplementary Table Supplementary Figures 1-14 and Supplementary Table 1

Supplementary Data 1 RNAseq comparison between bacteria in colonic mucus and colonic contents of *B. thetaiotaomicron* monocolonised mice. The table shows the raw data comparing *B. thetaiotaomicron* bacterial transcriptional pattern in colonic mucus and contents from the RNAseq data analysis by annotating all reads against *B. thetaiotaomicron* VPI 5482 reference genomic sequence. Abbreviations: Con, contents; muc, mucus. Normalisation to account for differences in overall library size was preformed prior to the test of differential gene expression.

Supplementary Data 2 RNAseq comparison between bacteria in colonic mucus and colonic contents of *E. coli* JM83 monocolonized mice. The table shows the raw data comparing *E. coli* JM83 bacterial transcriptional pattern in colonic mucus and contents from the RNAseq data analysis by annotating all reads against *E. coli* MG1655 reference genomic sequence. Abbreviations: Con, contents; muc, mucus. Normalisation to account for differences in overall library size was preformed prior to the test of differential gene expression.

Supplementary Data 3 STRING analysis. The genes in each STRING clusters are listed and detailed informations such as gene functional description, foldchange and padj are supplemented accordingly. The gene function description has been updated according to *B. thetaiotaomicron* genome reannotation (see Methods). Genes of *B. thetaiotaomicron* involved polysaccharide utilization loci (PULs) have been updated according to publications 24,26,27,28.

Supplementary Data 4 The table shows the raw data of Fig. 3d, e, f and Supplementary Figures 5 and 9 comparing transcription of *E. coli* and *B. thetaiotaomicron* bacterial gene in specific regulon, functional groups in colonic mucus and contents from either monocolonised or bicolonised mice. The gene lists have been reported by indicated previous publications. The statistic significance is ignored in these comparisons as the groups of individual genes are treated as an entirety.

Supplementary Data 5 The table shows the raw data comparing ion intensities in extraction from germ-free colonic mucus before and after *E. coli* growth. Average intensity is calculated from three biological replicates. The intensities of ions from interval time points are not shown. The orange colored wells indicate the standing rows show a statistical significance. The blue colored wells indicate the standing rows show a goodness of R2 fit greater than 0.8.

Supplementary Data 6 The table shows the raw data comparing ion intensities in extraction from germ-free colonic contents before and after *E. coli* growth. Average intensity is calculated from three biological replicates. The intensities of ions from interval time points are not shown. The orange colored wells indicate the standing rows show a statistical significance. The blue colored wells indicate the standing rows show a goodness of R2 fit greater than 0.8.

Supplementary Data 7 The table shows the raw data comparing ion intensities in extraction from germ-free colonic mucus before and after *B. thetaiotaomicron* growth. Average intensity is calculated from three biological replicates. The intensities of ions from interval time points are not shown. The orange colored wells indicate the standing rows show a statistical significance. The blue colored wells indicate the standing rows show a goodness of R2 fit greater than 0.8.

Supplementary Data 8 Comparison of ion intensities between colonic mucus of germ-free mice and monocolonised mice. The table shows the raw data comparing ion intensities in colonic mucus of either *E. coli/ B. thetaiotaomicron* monocolonised mice to germ-free mice. Average intensity is calculated from four biological replicates. Chemical taxonomy is cited from Human Metabolome database.

Supplementary Data 9 Comparison of ion intensities between colonic contents of germ-free mice and monocolonised mice. The table shows the raw data comparing ion intensities in colonic contents of either *E. coli* or *B. thetaiotaomicron* monocolonised mice to germ-free mice. Average intensity is calculated from four biological replicates. Chemical taxonomy is cited from Human Metabolome database.

Supplementary Data 10 RNAseq comparison between bacteria in colonic mucus and colonic contents of *B. thetaiotaomicron* in *E. coli* and *B. thetaiotaomicron* biocolonised mice. The table shows the raw data comparing *B. thetaiotaomicron* bacterial transcriptional pattern in colonic mucus and contents from the RNAseq data analysis by annotating all reads against *B. thetaiotaomicron* VPI-5482 reference genomic sequence. Abbreviations: Con, contents; muc, mucus. Normalisation to account for differences in overall library size was preformed prior to the test of differential gene expression.

Supplementary Data 11 RNAseq comparison between bacteria in colonic mucus and colonic contents of *E. coli* in *E. coli* and *B. thetaiotaomicron* biocolonised mice. The table shows the raw data comparing *E. coli* JM83 bacterial transcriptional pattern in colonic mucus and contents from the RNAseq data analysis by annotating all reads against *E. coli* MG1655 reference genomic sequence. Abbreviations: Con, contents; muc, mucus. Normalisation to account for differences in overall library size was preformed prior to the test of differential gene expression.

Supplementary Data 12 V5-V6 sequences of bacterial 16S rDNA of sDMDMm2 microbiota.

Supplementary Movie 1 Mobility of *E. coli* MG1655 *in vitro*. Time-lapse two-photon video showing the tumbling movement of GFP-expressing *E. coli* MG1655 *in vitro*. The display rate is 15 frames each second and the video contains one-second time intervals for five minutes. The scale bar indicates 20 μm length.

Supplementary Movie 2 Mobility of *E. coli* MG1655 in colonic outer mucus layer. Time-lapse two-photon video showing immobility of GFP expressing *E. coli* MG1655 in mucus of C57BL/6 mouse. The display rate is 15 frames each second and the video contains one-second time intervals for five minutes. The scale bar indicates 20 μm length.

Supplementary Movie 3 Mobility of *E. coli* MG1655 *in vitro* in colonic luminal liquid. Time-lapse confocal video showing the motility of GFP expressing E. coli MG1655 in luminal intestinal wash from J_H_^-/-^ mouse. The display rate is 15 frames each second and the video contains one-second time intervals for five minutes. The scale bar indicates 20 μm length.
