# Supplementary material for: The outer mucus layer hosts a distinct intestinal microbial niche
Source: Nat Commun. 2015 Sep 22;6:8292. doi: 10.1038/ncomms9292 (PMC4595636; doi:10.1038/ncomms9292)
Supplement: Supplementary Figures and Supplementary Table — Supplementary Figures 1-14 and Supplementary Table 1 [file ncomms9292-s1.pdf]

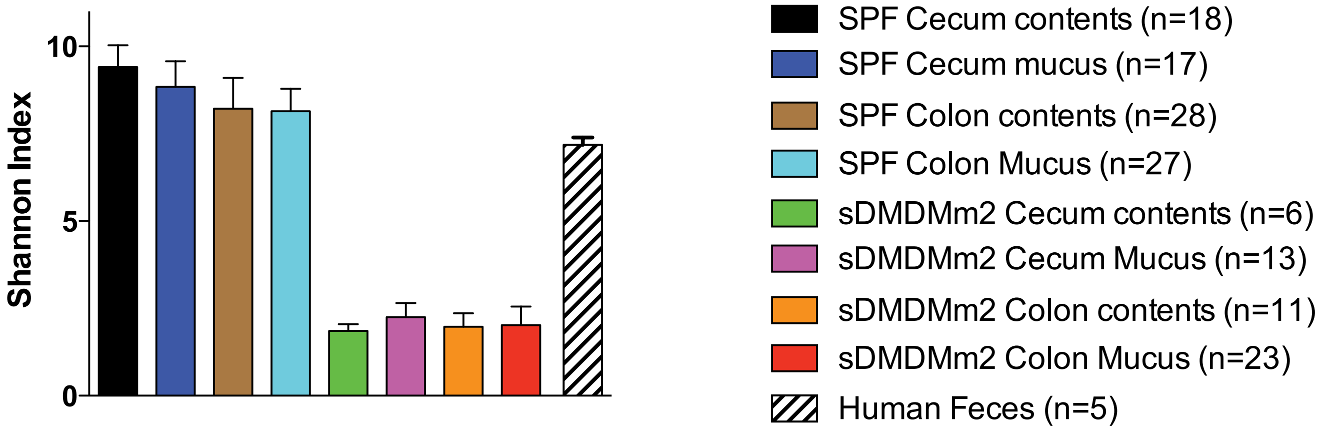


**Supplementary Figure 1 | Alpha diversity of different microbiotas.** The alpha diversity of different microbiotas in different niches was analyzed by 16S amplicon analysis. The Shannon index was calculated using the QIIME software pipeline. Bars and error bars represent the mean ± SD of the indicated number of samples**.** Specific pathogen free (SPF) mice were purchased Harlan Laboratories, Netherlands. Gnotobiotic mice with a stable defined moderately diverse microbiota (sDMDMm$_2$) were maintained at the clean mouse facility of the University of Bern. The sDMDMm2 microbiota is stable over time and consists of 12 defined bacterial species: *Bacteroides I48, Blautia YL58, Akkermansia YL44, Bacteroidales YL27, Ruminococcaceae KB18, Lactobacillus I49, Lachnospiraceae YL32, Erysipelotrichaceae I46, Enterococcus KB1, Flavonifractor YL31, Parasutterella YL45, Bifidobacterium YL2*. Human fecal samples were from healthy volunteers.

a

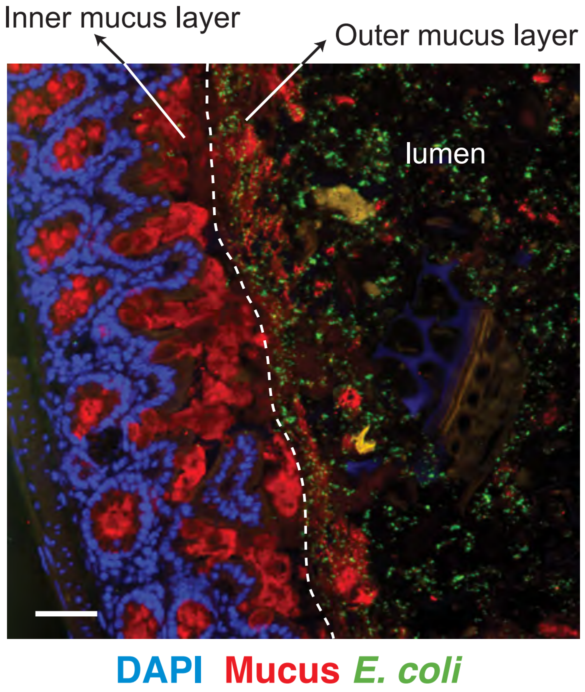


b

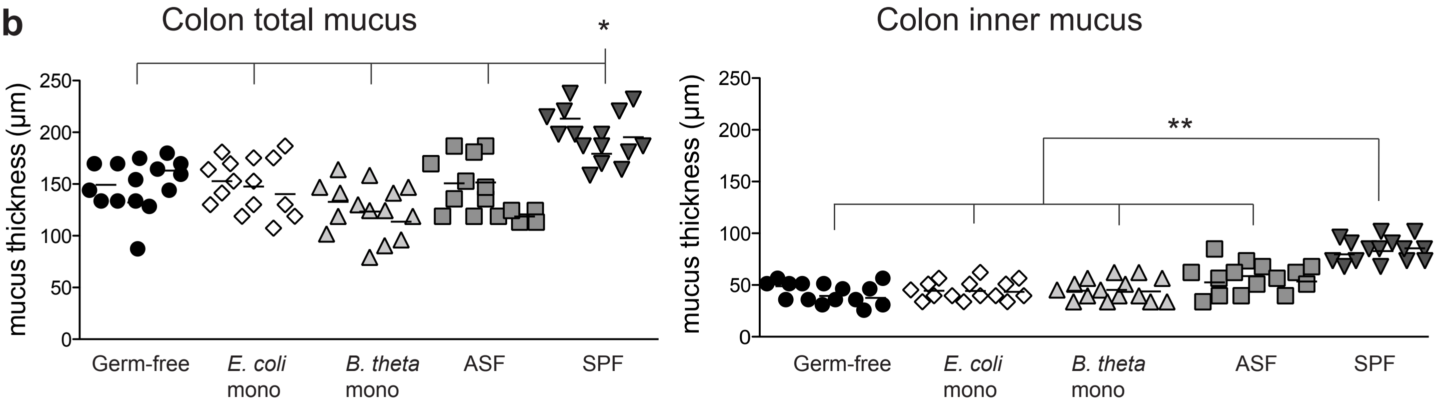


**Supplementary Figure 2 | Mucus-bacteria immunofluorescence staining and mucus *ex vivo* thickness measurements in the large intestines of C57BL/6 mice with differing colonisation statuses. a,** Sections of proximal colon of *E. coli* JM83 monocolonised mice were stained with polyclonal FITC-conjugated anti-*E. coli* mouse serum, polyclonal Alexa Fluor 546-conjugated anti-Muc2 antibody for mucus layer and DAPI (4',6'- diamidino-2-phenylindole). One representative image from three mice is shown.The scale bar indicates 50µm. **b,** Direct *ex-vivo* measurements of mucus thickness using a micromanipulator for the total (inner + outer layer, left panels) and inner layer of mucus (right panels) following removal of the outer mucus layer by aspiration in proximal colon segments in either germ-free, *E. coli* monocolonised, *B. thetaiotaomicron* monocolonised, ASF colonised or SPF colonised microbial hygiene statuses. Measurements were performed on five different sites per intestinal segment (n=3 individual mice per group). Unpaired t-test was performed and significance of SPF mice in relation to each of the other groups is shown as $p<0.05$ (*), $p<0.01$ (**).

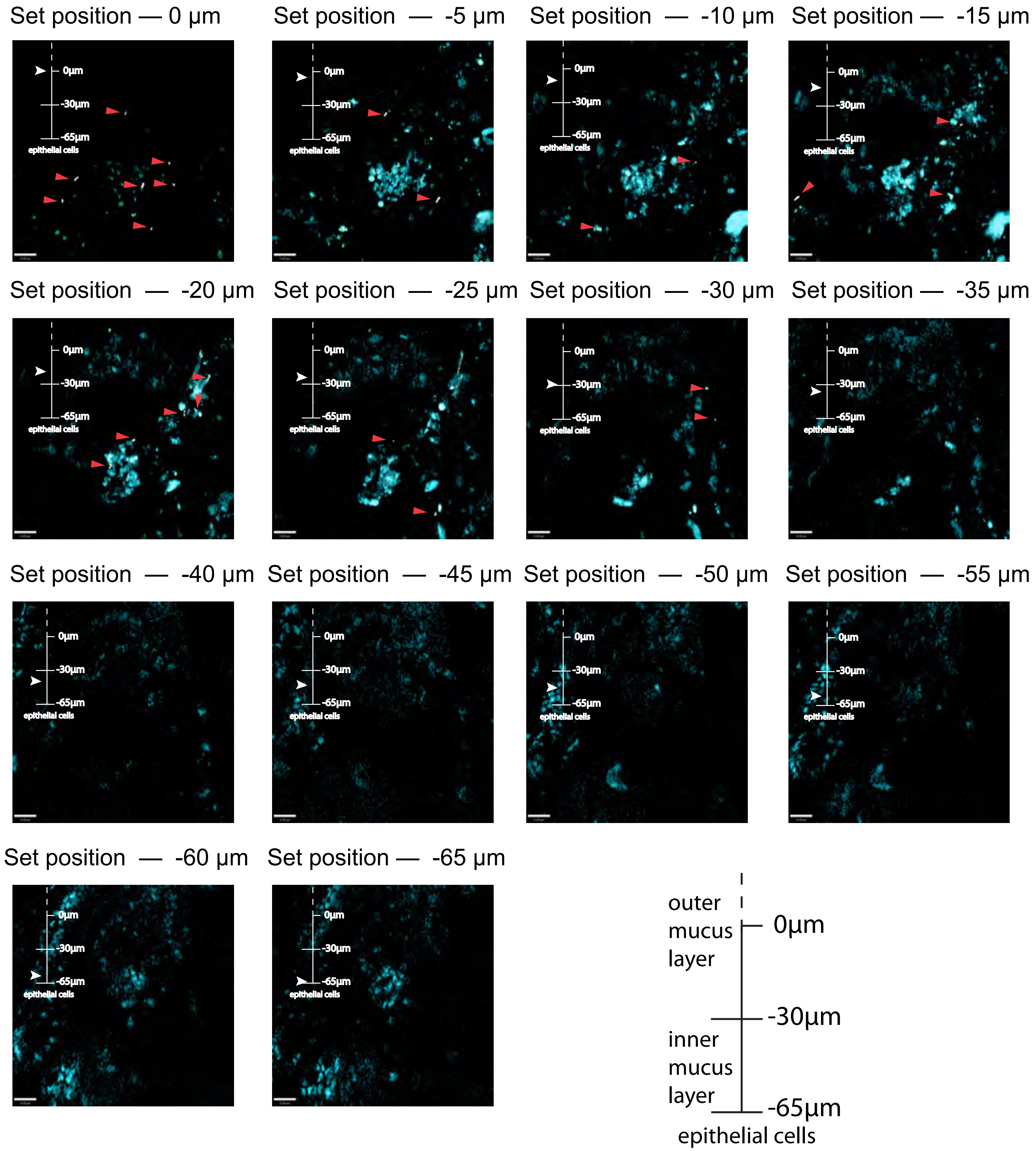


**Supplementary Figure 3 | Z-plane imaging of GFP-labeled *E. coli* in the mucus.** Colonic mucosa was isolated from *E. coli*-monocolonised mice. After longitudinal opening and contents removal, the section was immensed in water. A series of images were taken by 2-photon microscopy every 5µm in the Z-plane until the top of the intestinal villi. The red arrows indicate GFP-*E. coli:* for visualization the GFP channel has been displayed as white in the images. A schematic is shown on the bottom right to indicate the mucus structure.

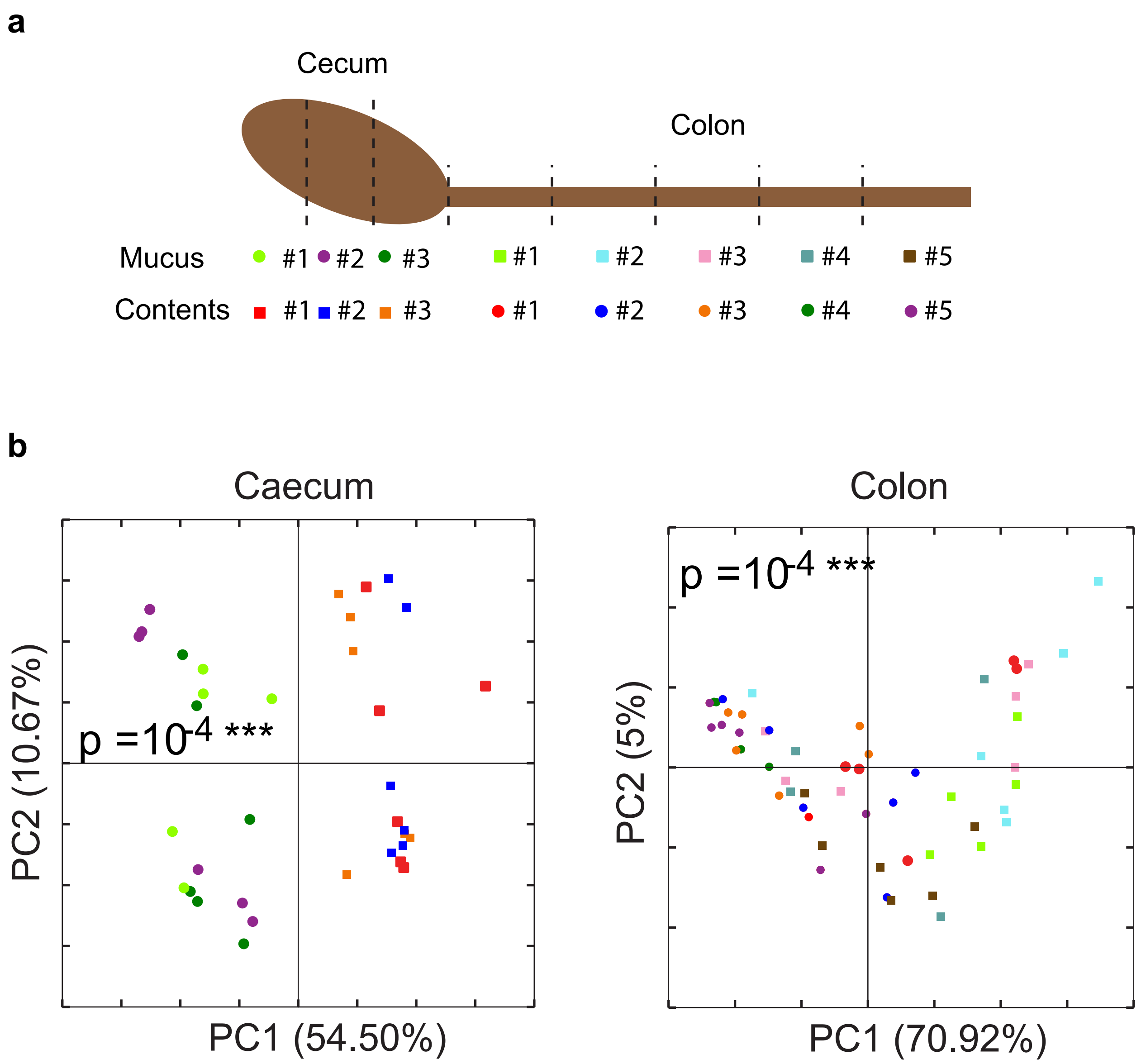


**Supplementary Figure 4 | Bacterial composition along the large intestine of SPF mice. a**, a schematic shows the experimental sample collection sites. **b**, The individual dots on principal coordinates analysis were colored seperately according to the sampling sites as indicated in (**a**). This figure displays the data in Figure 1 according to position along the length of the large intestine.

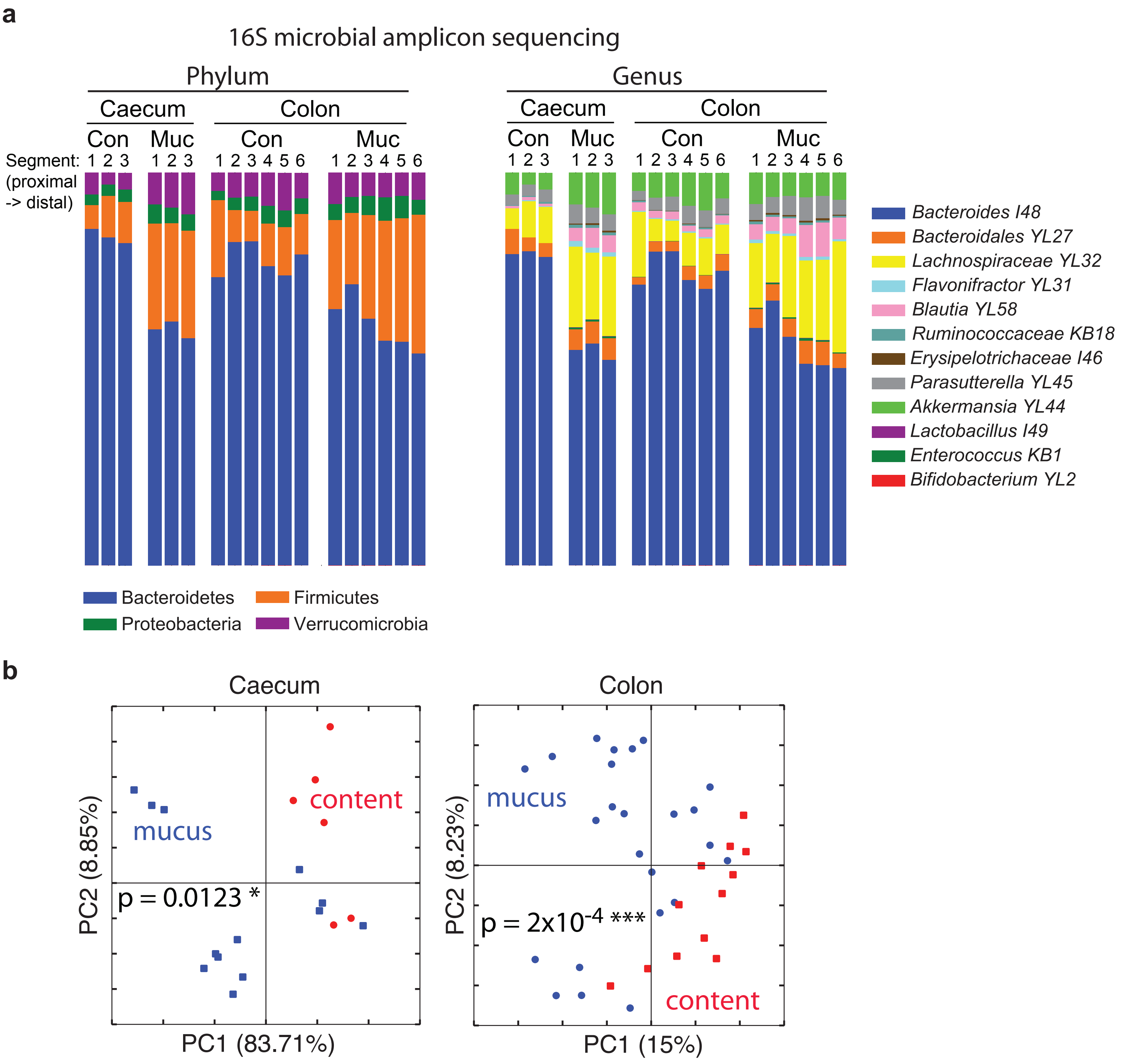


**Supplementary Figure 5 | Microbial communities in the mucus layer and luminal contents in the large intestine of stable defined medium density microbiota (sDMDM) gnotobiotic mice. a**, The microbial composition within the mucus layer and luminal content of different segments along the caecum and colon sDMDMm2 mice at both phyla (left panel) and species levels (right panel) were determined by 16S amplicon analysis. Representative bar graphs from a single analysis from different segments of the large intestine (see Supplementary Figure 4a) are shown. **b**, Principal coordinates analysis on weighted (caecum) or unweighted (colon) UniFrac distances with >500 reads/sample were performed on all OTUs. Each segment had colonic mucus for analysis, but some segments lacked colonic contents. p-values to determine the statistical significance of clustering were calculated using the Adonis method. Analysis was performed using QIIME 1.8.0.

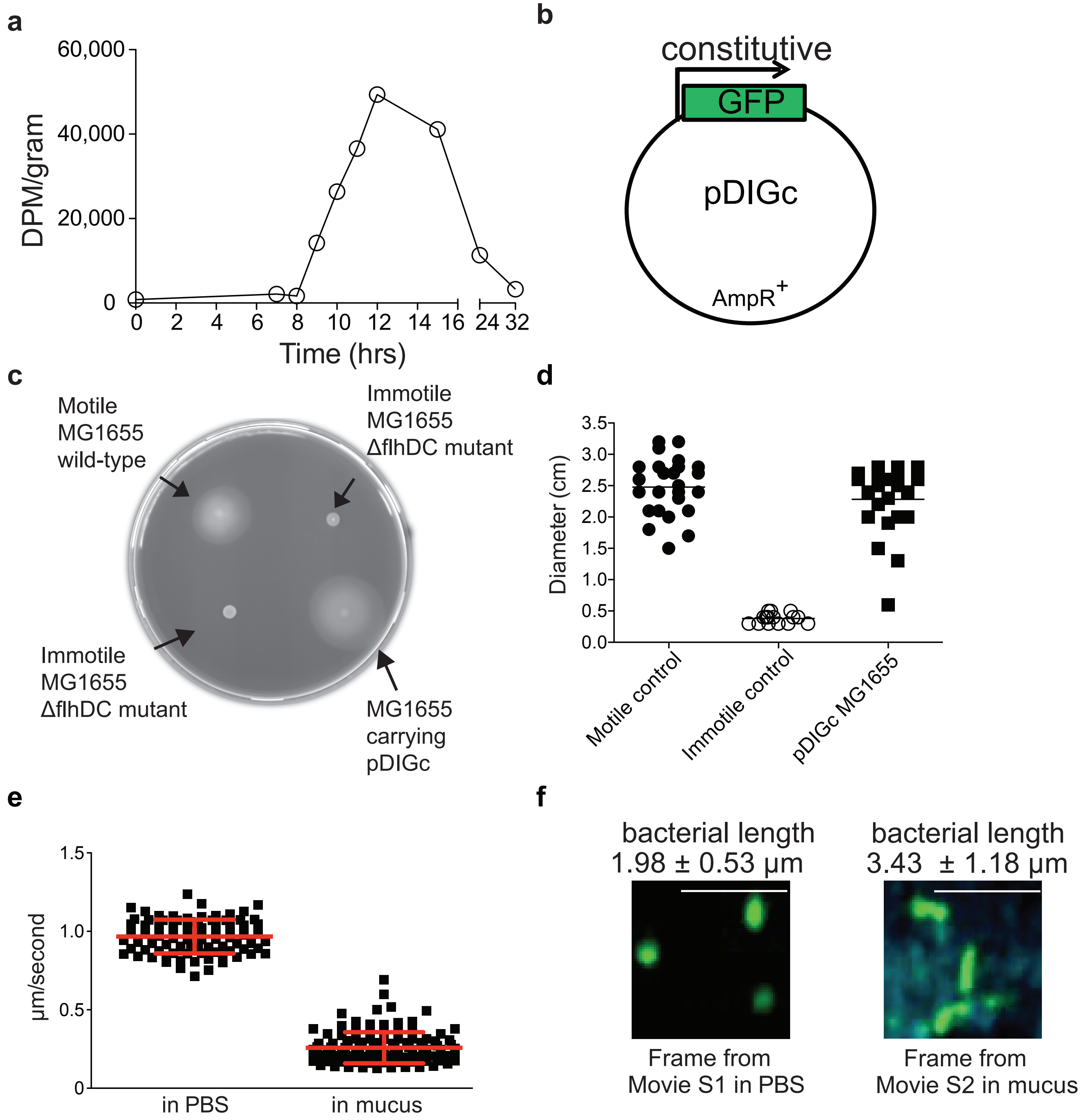


**Supplementary Figure 6 | Mucus turnover measurement and *in vitro* motility analysis of *E. coli* MG1655 carrying plasmid pDIGc. a,** ASF colonised mice were i.p. injected with 0.5 µCi/(gram body weight) [14C] N-acetylgalactosamine, and the levels of radioactivity (DPM) of colonic mucus and contents were monitored over time. The radioactivity in intestinal contents as background at each timepoint was substracted prior to DPM/gram mucus being plotted against that time point. Each dot indicates one mouse. The mucus turnover time was estimated after the 8 hour lag time before radiolabel had been incorporated into newly synthesised mucus that was released into the outer mucus layer from DPM per gram$^{max\ at\ t=11\sim12h}$ – DPM per gram$^{min\ at\ t=7\sim8h}$. Data represent two independent experiments. **b**, Plasmid map of pDIGc which contains a constitutive GFP expressing element. **c**, Motility assay of *E. coli* MG1655 carrying pDIGc, motile MG1655 (positive control) and immotile MG1655 ΔflhDC mutant (negative control for motility assay). Bacteria were inoculated on a semi-solid tryptone motility agar (LB broth with 2% Bacto agar) and grown at room temperature for 18 hours. Diameters of bacterial colonies are shown in (**d**). **e,** The average track velocity (µm/second) of bacteria tumbling in PBS or in colonic mucus from GFP - *E. coli* colonised mice were measured using time-lapse two-photon microscopy over a time span of 5 minutes. **f**, Representative frames were taken from bacteria tumbling *in vitro* in PBS (Supplementary Movie 1) or *ex vivo* in colonic mucus (Supplementary Movie 2) and the bacterial length values (mean±SD) were measured from n= >30 images on each frame. The scale bars indicate 10µm.

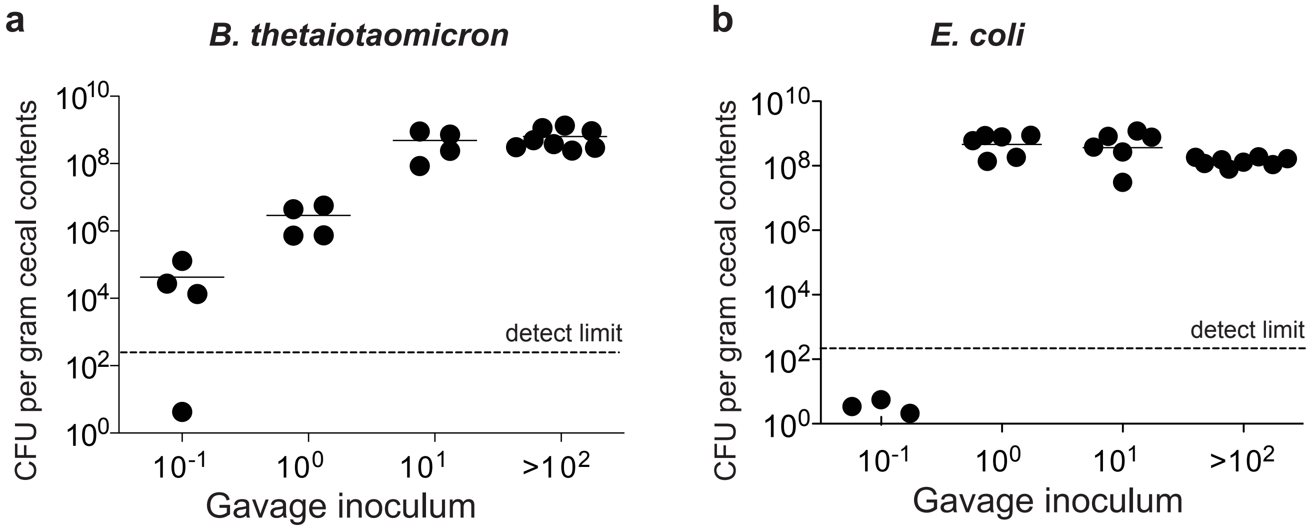


**Supplementary Figure 7 | Sensitivity of germ-free mice to bacterial colonisation.** Germ-free C57BL/6 mice were gavaged with a titrated dose of $10^{-1}$, $10^{0}$, $10^{1}$, and ≥ $10^{2}$ CFU of (**a**) *B. thetaiotaomicron* or (**b**) *E. coli.* The total CFU per gram cecal contents was determined after 18 hours of colonisation when steady-state had been achieved with a dose of ≥$10^{1}$ CFU. Data from one (*B. thetaiotaomicron*) or three (*E. coli*) independent experiments are shown. Each datapoint represents one mouse.

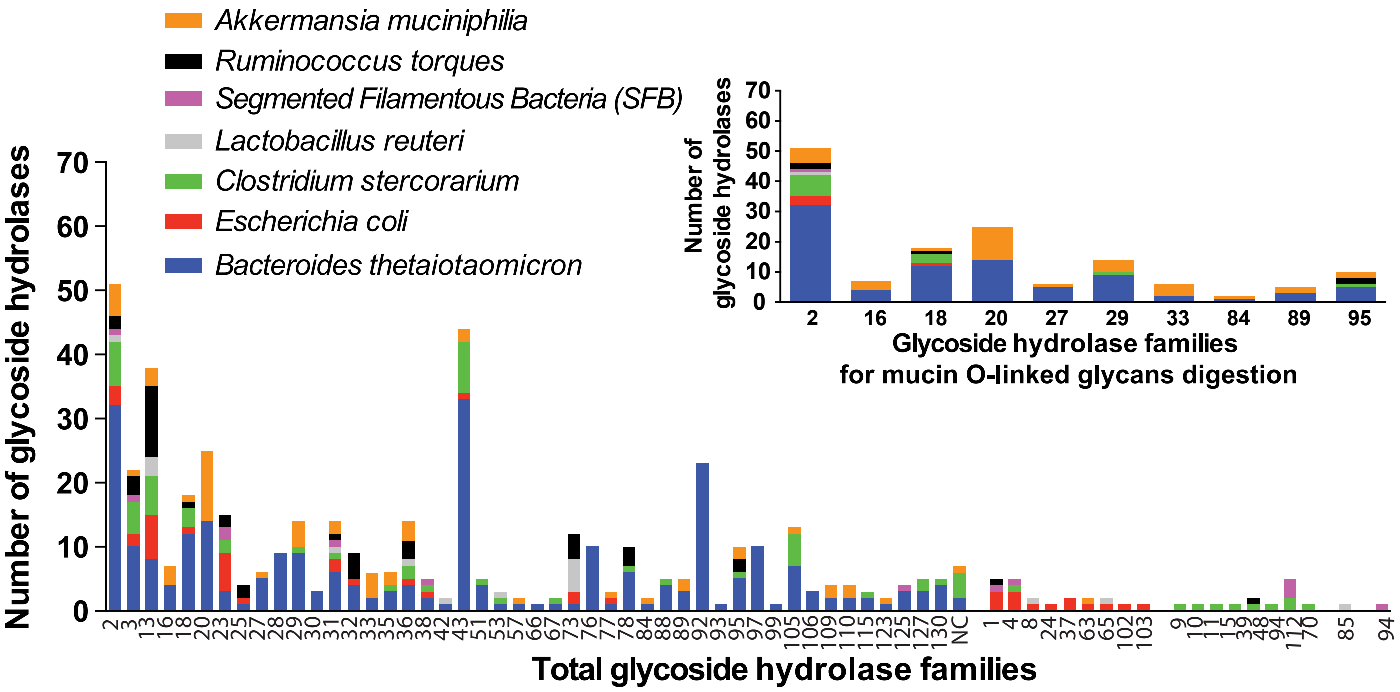


**Supplementary Figure 8 | The distribution of glycoside hydrolases (GH) in seven species of intestinal bacteria,** The glycoside hydrolases contained by each bacterial species are classified into glycosidase families summarized on the CAZY database (www.cazy.org). The GH families are listed on the X-axis and plotted against the number of glycoside hydrolases of each bacterial species. The upper right inset highlights the glycoside hydrolase families specific for degradation of mucin O-linked glycans.

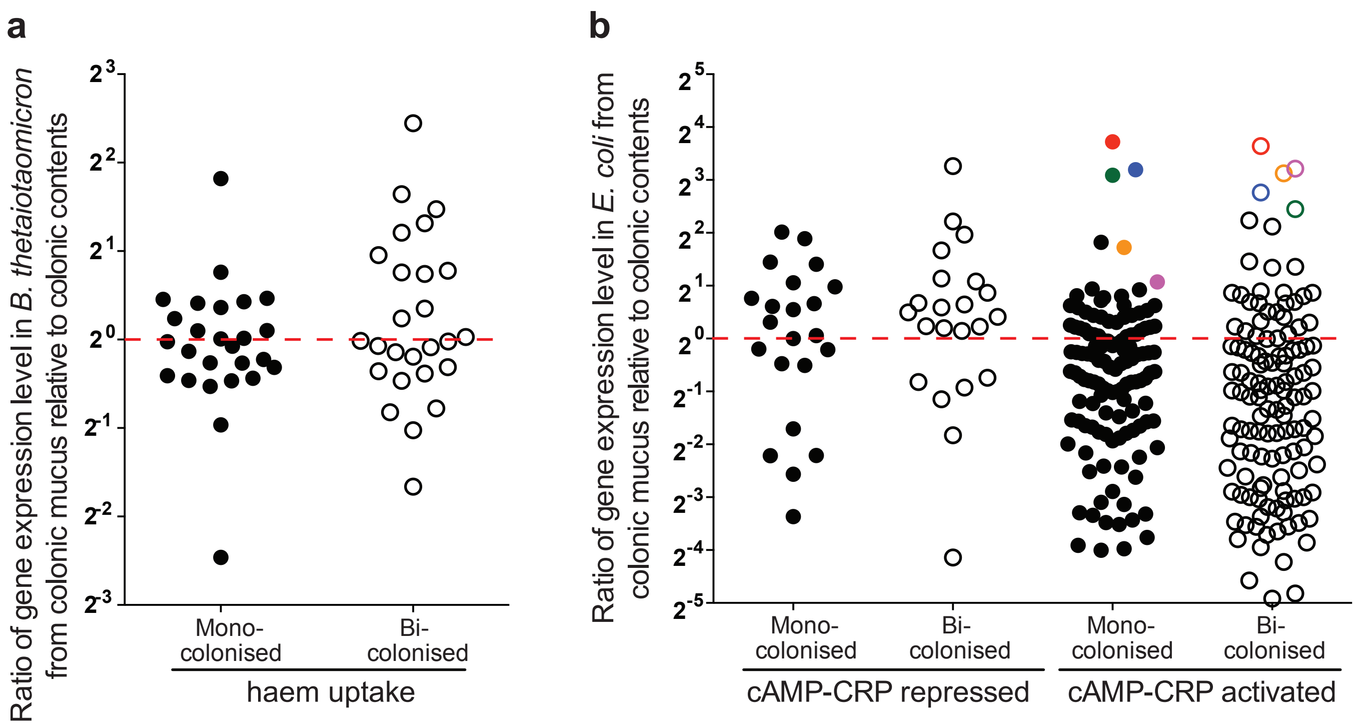


**Supplementary Figure 9 | Gene expression ratios between colonic mucus and luminal contents belonging to indicated regulons or functional groups**. Mucus/content ratios of transcripts of genes involved in (**a**) haem uptake genes of *B. thetaiotaomicron;* or (**b**) subject to the cAMP-CRP regulon [35] of *E. coli* as defined previously. The expression ratios of all known genes in each group are plotted. The main discoordinated genes were highlighted as ●○ entC (iron uptake, fur regulon) and ●○ fepA (iron-enterobactin transporter, fur regulon), which are also regulated by Fur as shown in Fig. 4a;○●pdhR-aceEF (pyruvate dehydrogenase complex), also regulated by FNR/ArcA as shown in Fig. 4c; ●○ SodA (superoxide dismutase, Mn) and ●○ MarR (multidrug and toxic compounds efflux regulator). The annotations for all displayed dots are documented in Supplementary Data 4. The red dotted line indicates unity of expression ratios between colonic mucus and contents.

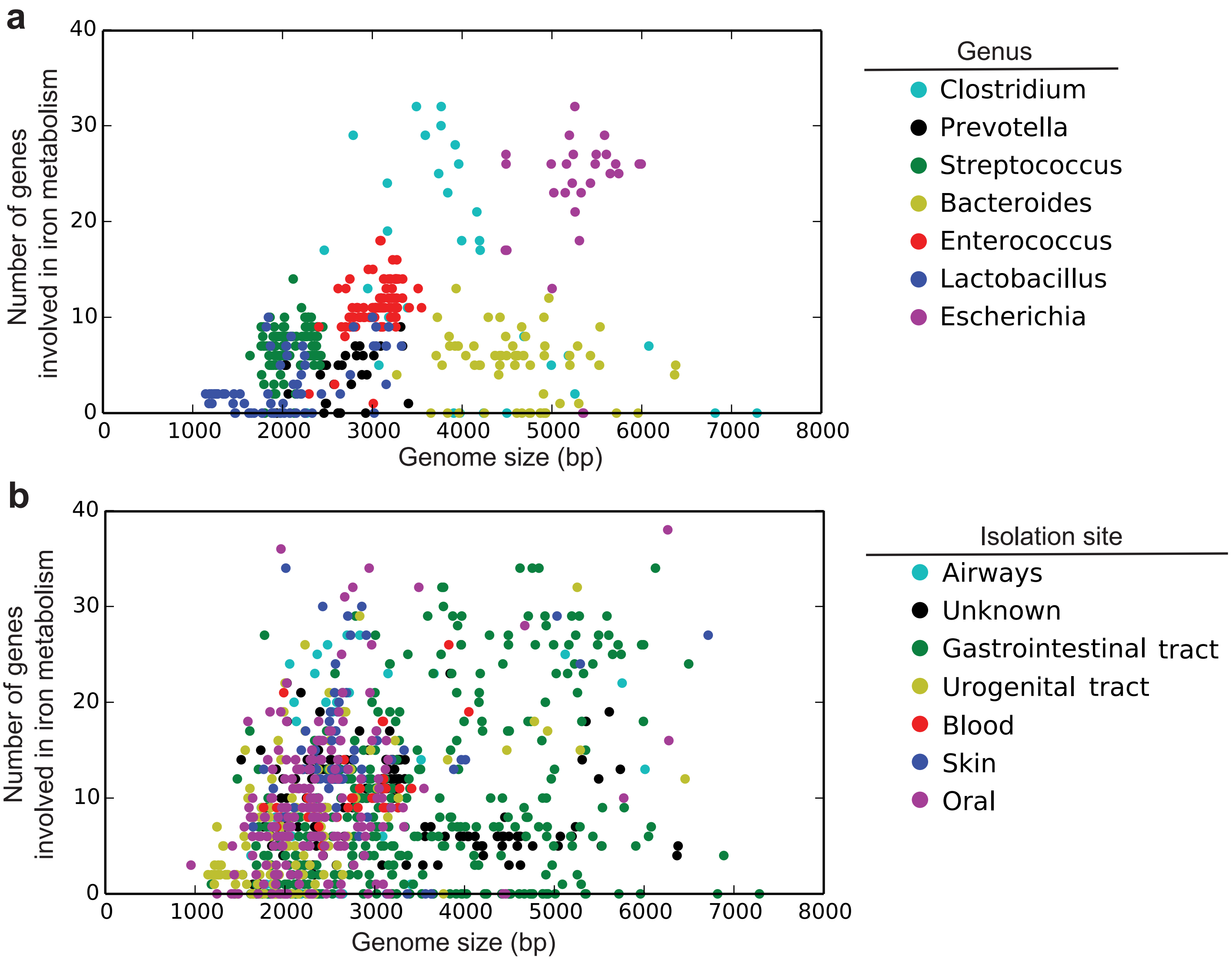


**Supplementary Figure 10 | Genomic capability of iron metabolism among bacterial kingdom.** **a,** Distribution per genus of genes involved in iron metabolism in each bacterial genome. The bacteria of the Clostridium genus use haem as an iron source hence their smaller repertoire of enzymes for iron metabolism (eg. acquisition, transport etc...) compared to Escherichia bacteria which are dependent on ferric iron. **b**, Distribution per isolation site of genes involved in iron metabolism in each bacterial genome. Bacteria isolated from the gastrointestinal tract present different strategies regarding iron, either investing in a significant number of genes for iron metabolism or almost none. For both panels, the number of genes is plotted against the genome size of each bacterial genome from the Human Microbiome Project dataset (number of genomes included at the time of analysis was 1290).

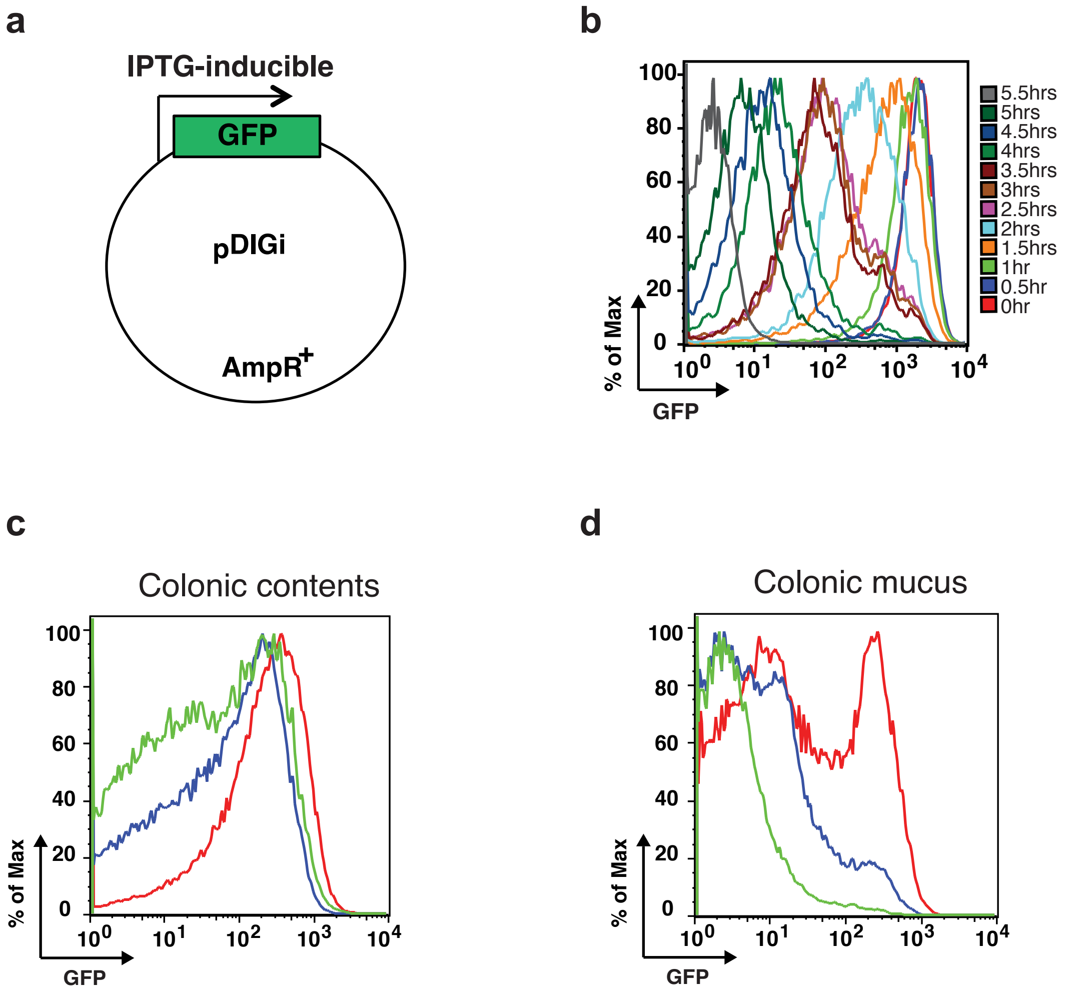


**Supplementary Figure 11 | *E. coli* replication in colonic mucus and luminal contents at a single cell level. a**, Schematic of the plasmid map for pDIGi which contains an IPTG-inducible GFP expression element. **b**, *E. coli* MG1655 carrying pDIGi were grown in non-permissive culture conditions (no IPTG) for 5.5 hours, and aliquots of bacteria at indicated time points were analysed for GFP fluorescence intensity by flow cytometry: this data was used to generate calibration cuves of division number against loss of mean fluorescence intensity. Germ-free C57BL/6 mice were colonised with a saturating dose of GFP labeled *E. coli* MG1655-pDIGi for 7 hours (red histogram), 9 hours (blue histogram), or 11 hours (green histogram), when both (**c**) colonic contents and (**d**) mucus were isolated and GFP-labeled bacteria were visualised by flow cytometry. Data are representative of three independent experiments.

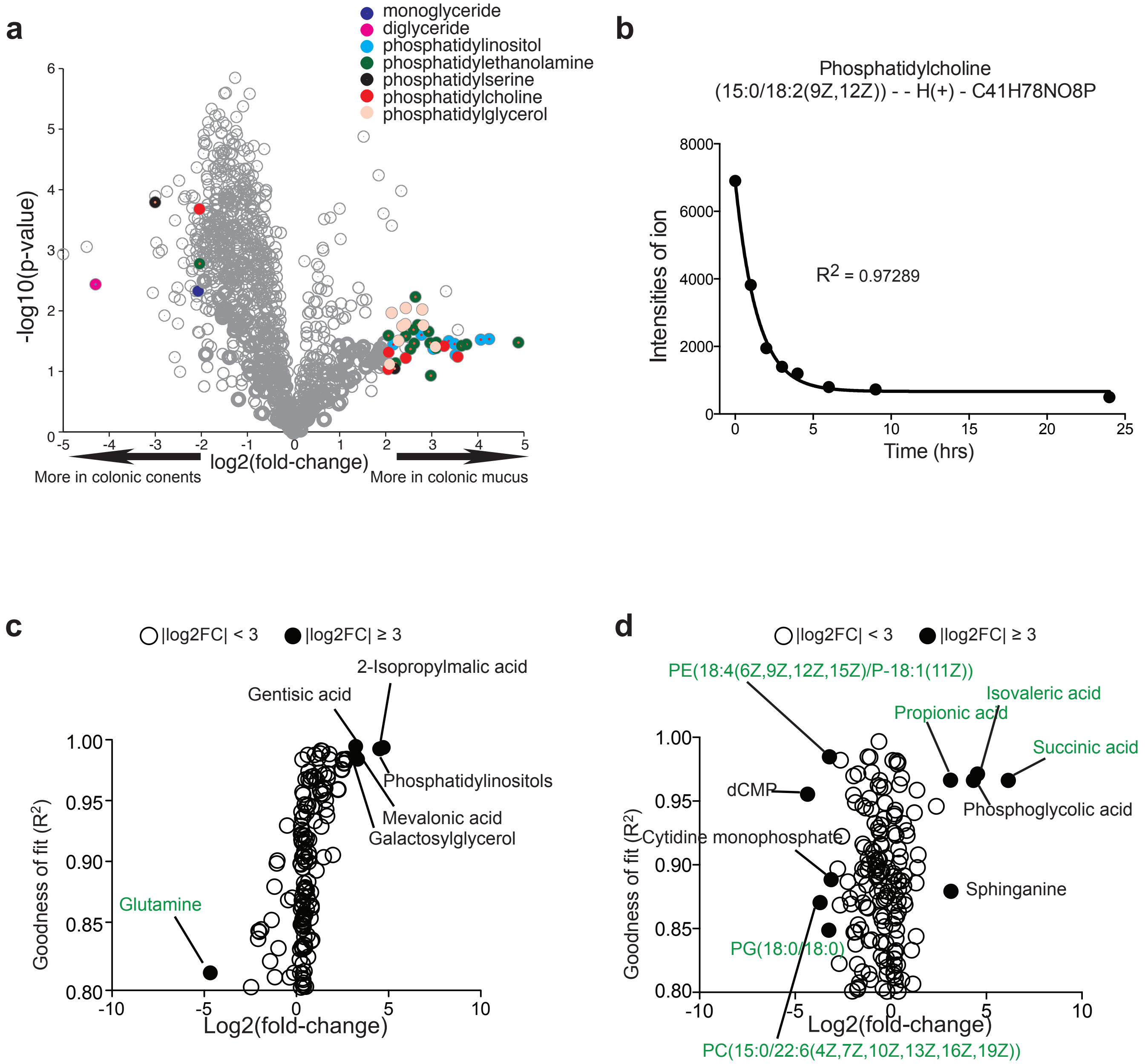


**Supplementary Figure 12 | Metabolic comparison between colonic mucus/contents and *E. coli* and *B. thetaiotaomicron in vitro* consumption assay. a,** Colonic mucus and luminal contents were isolated from *E. coli* monocolonised mice. The metabolites were extracted by hot water and measured by non-targeted mass spectrometry. All phospholipids and glyceride compounds (each with different defined acyl chains) meeting the criteria of (log2(fold-change) ≥ 2 and p-value ≤ 0.05) are highlighted by indicated colors. A complete dataset with molecular annotations is given in Supplementary Data 5. Bacteria were cultured on *ex vivo* extracts collected from (**b** and **d**) colonic mucus or (**c**) luminal contents of germ-free mice and cultures were sampled at several time points. **b**, A representative goodness of fit $R^2$ is shown to explain the relation between $R^2$ score and continuous metablolite consumption. The consumed and secreted metabolites were identified comparing metabolic pattern before and after (**c**) *E. coli* on colonic contents or (**d**) *B. thetaiotaomicron* growth on colonic mucus. Complete datasets with molecular annotations were given in Supplementary Data 6 for (**c**) and Supplementary Data 7 for (**d**).

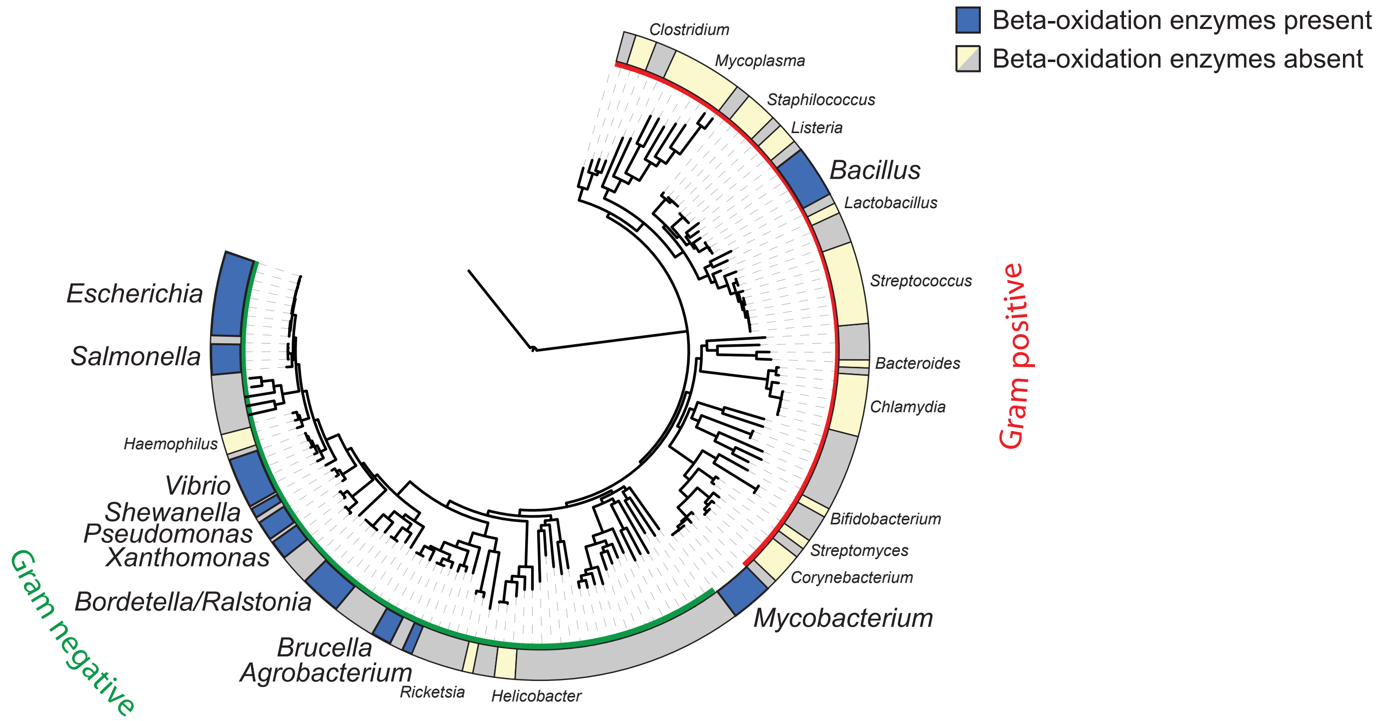


**Supplementary Figure 13 | Genomic capability of fatty acid degradation among bacterial kingdom.** Phylogenetic distribution of the 4 core enzymes implicated in the β-oxidation pathway. Genera highlighted in blue have conserved all 4 enzymes. All other genera have lost part or all enzymes implicated in this pathway. Indicated in light yellow are other major genera of the bacterial tree of life.

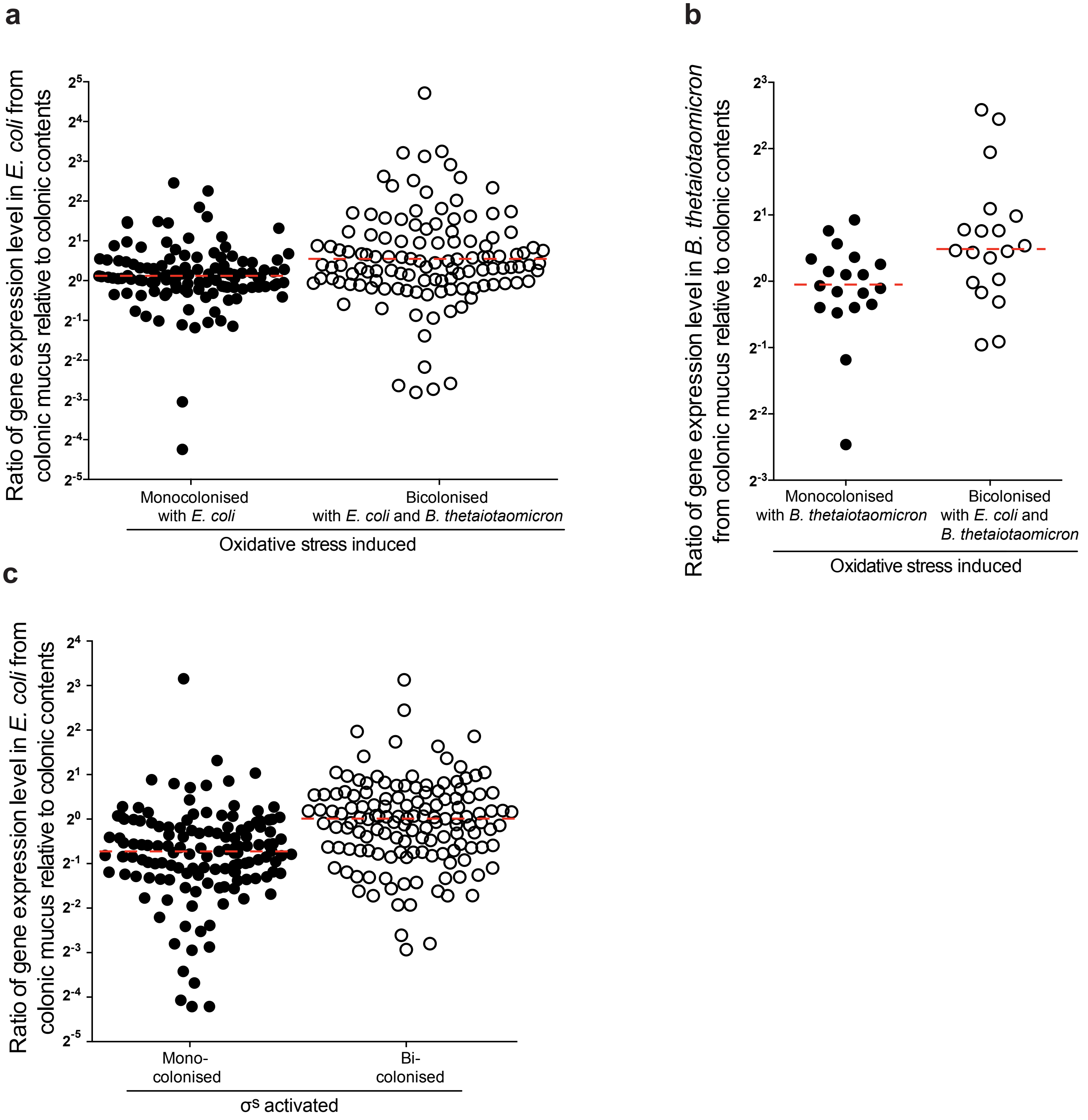


**Supplementary Figure 14 | Functional grouping of gene comparison analysis.** Oxidative stress-induced genes of (**a**) *E. coli* or (**b**) *B. thetaiotaomicron* were compared between colonic mucus and contents from *E. coli* and *B. thetaiotaomicron* bicolonised mice. **c**, $\sigma^S$ activated genes of *E. coli* [41] were compared between colonic mucus and contents from *E. coli* monocolonised mice and *E. coli* and *B. thetaiotaomicron* bicolonised mice. The red dashed lines indicate the geometric mean of each group. The complete annotations of all displayed dots for (**a**) (**b**) (**c**) are listed in Supplementary Data 4.

| Supplementary Table 1 \| Comparisons of dynamics of *E. coli* and *B. thetaiotaomicron* | | |
|---|---|---|
| | *E. coli* | *B. thetaiotaomicron* |
| Maximal replication rate in empty gut (< $10^4$ CFU inoculum) | 0.65 ± 0.01 (hrs) | 0.81 ± 0.03 (hrs) |
| Steady-state replication rate in colonic mucus (monocolonisation) | 2.86 ± 0.26 (hrs) | 3.14 ± 0.42 (hrs) |
| Steady-state replication rate in colonic contents (monocolonisation) | 7.16 ± 1.85 (hrs) | 3.54 ± 0.54 (hrs) |
| Steady-state replication rate in colonic mucus (bicolonisation) | 4.39 ± 0.67 (hrs) | |
| Steady state replication rate in colonic contents (bicolonisation) | 12.89 ± 6.47 (hrs) | |
| CFU in the whole gastrointestinal tract of monocolonised mice | $3.55 \pm 1.75 \times 10^9$ | $3.26 \pm 1.64 \times 10^{10}$ |
| Every 24hrs shedding CFU/mouse by feces | $3.52 \pm 1.66 \times 10^9$ | $6.22 \pm 4.30 \times 10^{10}$ |
| Sensitivity of germ-free mice to colonisation | < 10 bacteria | < 10 bacteria |
